# Sarcosine-Based Pharmacokinetic Optimization and Fluorescent Dye Library Evaluation of Dual-Labeled PSMA Inhibitors for Fluorescence-Guided Surgery

**DOI:** 10.3390/ph19081187

**Published:** 2026-07-29

**Authors:** Paul Minges, Jessica Matthias, Lisa-Charlotte Domogalla, Björn Thomas, Nils Steinacker, Nawal Ayada Amgar, Holger Müller, Antje Dietzel-Schaarschmidt, Philipp T. Meyer, Matthias Eder, Ann-Christin Eder

**Affiliations:** 1 Department of Nuclear Medicine, University Medical Center Freiburg, Faculty of Medicine, University of Freiburg, 79106 Freiburg, Germany; paul.minges@uniklinik-freiburg.de (P.M.);; 2 Division of Radiopharmaceutical Development, German Consortium for Translational Cancer Research (DKTK), Partner Site Freiburg and German Cancer Research Center (DKFZ), 69120 Heidelberg, Germany; 3 Department of Biology, University of Freiburg, 79104 Freiburg, Germany; 4 Abberior GmbH, 37077 Göttingen, Germany; 5 XION GmbH, 13127 Berlin, Germany; 6 Henke-Sass, Wolf GmbH, 78532 Tuttlingen, Germany

**Keywords:** PSMA, prostate cancer, fluorescence-guided surgery, dual-labeled, Theranostics, small peptide

## Abstract

**Objectives**: Fluorescence-guided surgery (FGS) targeting prostate-specific membrane antigen (PSMA) holds promise for improving surgical precision in prostate cancer. Since conjugation of fluorescent dyes to targeting vectors can substantially alter pharmacokinetic properties, we systematically evaluated a library of fluorescent dyes conjugated to a PSMA-617-derived scaffold incorporating sarcosine-based spacers to identify candidates with favorable biodistribution and optical profiles for clinical translation. **Methods**: Nineteen fluorescent dyes spanning NIR, large Stokes shift, and STED-compatible categories were conjugated to a dual-labeled PSMA-617-derived precursor (Glu-urea-Lys-2Nal-TXA-Sar_10_-Lys(DOTA)-Sar_5_-βAla; hereafter DP). Compounds were radiolabeled with ^68^Ga or ^177^Lu and characterized for serum stability, lipophilicity, binding affinity, and internalization in LNCaP^PSMA+^ cells. In vivo pharmacokinetics were assessed in LNCaP xenograft-bearing BALB/c nu/nu mice by µPET/MRI (1 and 2 h p.i., 500 pmol ^68^Ga), organ distribution (0.5, 1, and 2 h p.i., 60 pmol ^177^Lu), and clinical-grade endoscopic fluorescence imaging. **Results**: All conjugates retained hydrophilic character (logD: −3.72 to −1.79), low nanomolar binding affinity (K_i_: 18–87 nM), and high serum stability (94–100% intact at 24 h). Despite comparable in vitro properties, dye conjugation markedly influenced in vivo pharmacokinetics: tumor uptake at 2 h p.i. ranged from 1 to 23%ID/g and kidney accumulation from 3 to 82%ID/g. Visible-range dyes exhibited faster renal washout within the imaging window and higher tumor-to-background contrast than NIR fluorophores. Fluorescence signal intensity did not correlate with radiotracer-derived uptake, underscoring the importance of dye-specific photophysical properties. **Conclusions**: DP-12 (SulfoCy5), DP-15 (Alexa Fluor 647), and DP-18 (Tide Fluor 5WS) were identified as lead candidates combining favorable pharmacokinetics with strong fluorescence contrast, warranting further evaluation toward fluorescence-guided prostate cancer surgery.

## 1. Introduction

Prostate cancer (PC) is one of the most frequently diagnosed malignancies in men worldwide [1], and radical prostatectomy (RP) remains a primary curative treatment option for early-stage, localized disease [2]. However, achieving complete tumor resection while preserving surrounding functional structures poses a significant surgical challenge. It was reported that positive surgical margins still occur in 16% of cases after robotic-assisted RP [3] and are associated with increased risk of biochemical recurrence, often necessitating adjuvant therapy [4,5]. Real-time intraoperative tumor visualization through fluorescence-guided surgery (FGS) could improve identification of PC tissue and surgical precision, and reduce positive surgical margin and, ultimately, relapse rates [6].

Prostate-specific membrane antigen (PSMA) has emerged as a highly validated molecular target for PC [7]. PSMA-targeted radiotracers such as [^68^Ga]Ga-PSMA-11 and [^177^Lu]Lu-PSMA-617 have been successfully translated into clinical practice for diagnostic imaging and radioligand therapy (RLT), respectively [8,9,10]. The clinical success of these compounds has prompted efforts to develop dual-labeled PSMA inhibitors that combine radioactive and fluorescent moieties, enabling both preoperative staging via positron emission tomography (PET) imaging and intraoperative FGS [11]. Depending on the radionuclide used, such compounds may additionally support radio-guided surgery (RGS), as established in clinical practice with ^99m^Tc-PSMA-I&S [12]. The modular design presented here enables preoperative staging (^68^Ga, ^111^In) and intraoperative FGS/RGS within a single compound platform, with potential extension to therapeutic applications where clinically indicated. Consolidating preoperative imaging and intraoperative guidance into a single same-day session also limits the number of separate radioactive administrations required, which is favorable for both patient management and tracer handling.

Current dual-labeled approaches for FGS predominantly employ near-infrared (NIR) fluorophores such as indocyanine green (ICG) or IRDye800CW, based on their favorable tissue penetration and reduced autofluorescence interference [13,14]. However, conjugation of fluorescent dyes, which can contribute 25–50% of the total molecular weight of small peptide-based tracers, may substantially alter pharmacokinetic properties, including blood clearance, tumor accumulation, and renal retention [15]. Despite this, systematic comparisons across diverse fluorescent dye classes remain limited.

In this study, we evaluated a library of 19 fluorescent dyes conjugated to a PSMA-617-derived scaffold. The library was designed to span three distinct dye categories: near-infrared (NIR) dyes, selected for their favorable tissue penetration and reduced autofluorescence interference; large Stokes shift dyes, included to assess whether reduced spectral overlap between excitation and emission improves imaging contrast; and stimulated emission depletion (STED)-compatible dyes, chosen for their superior brightness and photostability for intraoperative visualization. Through comprehensive in vitro characterization, in vivo imaging, biodistribution analysis, and fluorescence imaging using a clinical-grade endoscopic system, we aimed to identify candidates with favorable optical and pharmacokinetic profiles suitable for clinical translation in fluorescence-guided PC surgery.

## 2. Results

### 2.1. Evaluation Strategy

Compounds were first required to meet basic in vitro criteria: high serum stability, nanomolar PSMA-binding affinity, and PSMA-specific internalization. The framework was deliberately scoped to compounds compatible with a same-day surgical workflow. In vivo, we prioritized compounds in which kidney and background organ retention had decreased sufficiently by the surgical imaging window (1–2 h p.i.) to avoid dominating the field of view, while tumor uptake remained as high as possible within the library. Compounds were further required to show no specific accumulation in non-target tissues at 2 h p.i. Finally, candidates intended for direct clinical translation needed to be spectrally compatible with the available endoscopic imaging hardware, which is currently optimized for the SulfoCy5 spectral range. Compounds outside this spectral window were nevertheless included in the in vivo characterization, since future advances in FGS hardware may extend the accessible range and our data would provide a starting point for such developments.

### 2.2. Chemistry and Radiochemistry

A library of 19 dual-labeled compounds was successfully synthesized (Table 1). All conjugates were obtained with high chemical purity (>95%), as confirmed by analytical RP-HPLC [Supplementary Figure S1]. The molecular identity of each compound was verified by MALDI-TOF-MS, with all observed masses matching the calculated values, detected as either [M + H]^+^ or [M + Na]^+^ adducts [Supplementary Figure S2].

**Figure 1:**
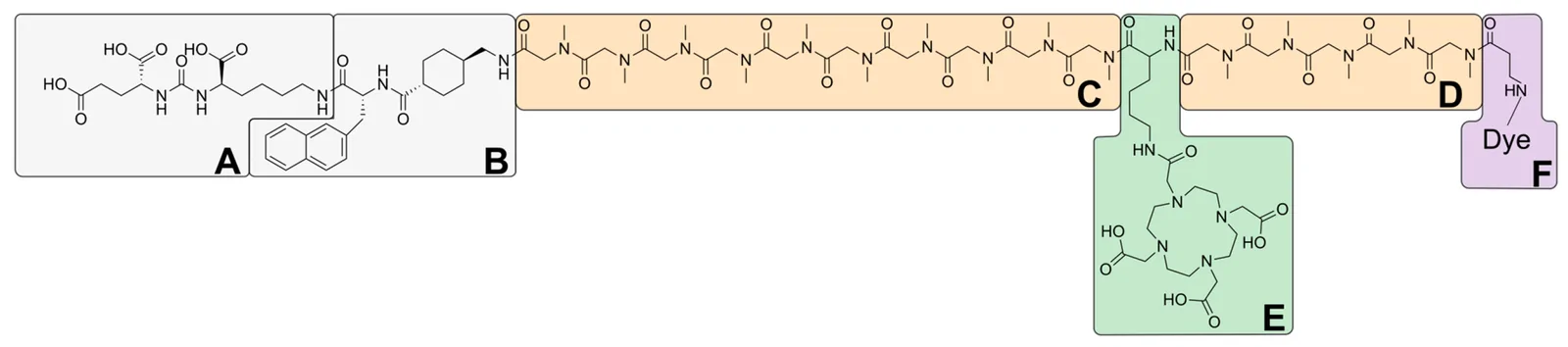
Modular structure of the dual-labeled PSMA-targeting precursor DP and its dye conjugates (DP-#). (**A**) Glutamate–urea–lysine PSMA-binding motif (grey); (**B**) 2-naphthylalanine-tranexamic acid linker (grey); (**C**) 10-mer sarcosine spacer (orange); (**D**) 5-mer sarcosine spacer (orange); (**E**) lysine branching unit with DOTA chelator for radiolabeling (green); (**F**) β-alanine with variable fluorescent dye moiety (purple), conjugated via NHS-ester chemistry. Dyes correspond to Table 1.

The dual-labeled compounds were based on the established glutamate–urea–lysine (EuK) PSMA-binding motif derived from PSMA-617. To minimize potential steric interference between the fluorescent dye and the target-binding domain, sarcosine-based spacers were introduced, as shown in Figure 1. Fluorescent dyes were conjugated through NHS-esters to the PSMA-617-derived peptide scaffold (2307.59 g/mol).

Radiolabeling with both ^68^Ga and ^177^Lu was achieved under standardized conditions, yielding radiochemical purities exceeding 99% for all compounds, in accordance with published consensus guidelines [16]. One compound (DP-24) exhibited increased surface adsorption following dye conjugation, requiring addition of 0.05% Tween-20 to achieve consistent labeling efficiency.

### 2.3. In Vitro Evaluation

The lipophilicity (logD at pH 7.4) of the compound library ranged from −3.72 to −1.79, confirming the hydrophilic character of all tested conjugates (Table 2).

Serum stability revealed that most compounds exhibited high stability in human serum, with 94–100% of the intact compound remaining after 24 h of incubation and only a gradual decrease observed up to 72 h. In murine serum, degradation was more pronounced within the first 24 h, with intact compound levels ranging from 77 to 100% and accelerated degradation towards 72 h. Notably, DP-4, DP-11 and DP-13 displayed accelerated degradation in both human and murine serum from the onset of incubation. DP-17 showed high instability and was excluded from further experiments (Table 2).

Competitive cell-binding assays demonstrated binding affinities against PSMA in the low nanomolar range, with K_i_ values spanning from 18.03 nM to 86.89 nM (Table 2) (precursor without dye has K_i_ = 13.2 nM) [Supplementary Figure S3].

Internalization assays further confirmed specific binding to the PSMA epitope. Surface-bound activity ranged from 0.07 to 0.57%AA/105 cells, while internalized activity ranged from 0.07 to 0.79%AA/10^5^ cells (Table 2) (precursor without dye has 2.2%AA/10^5^ cells). Specificity was verified by co-incubation with the PSMA inhibitor 2-PMPA, which effectively blocked both surface binding and internalization. Additionally, PSMA-mediated endocytosis was confirmed by parallel incubation at 4 °C, under which ATP-dependent internalization was inhibited while ATP-independent surface binding was preserved [Supplementary Figure S4].

### 2.4. In Vivo Evaluation

#### 2.4.1.   PET/MR Imaging

**Figure 2:**
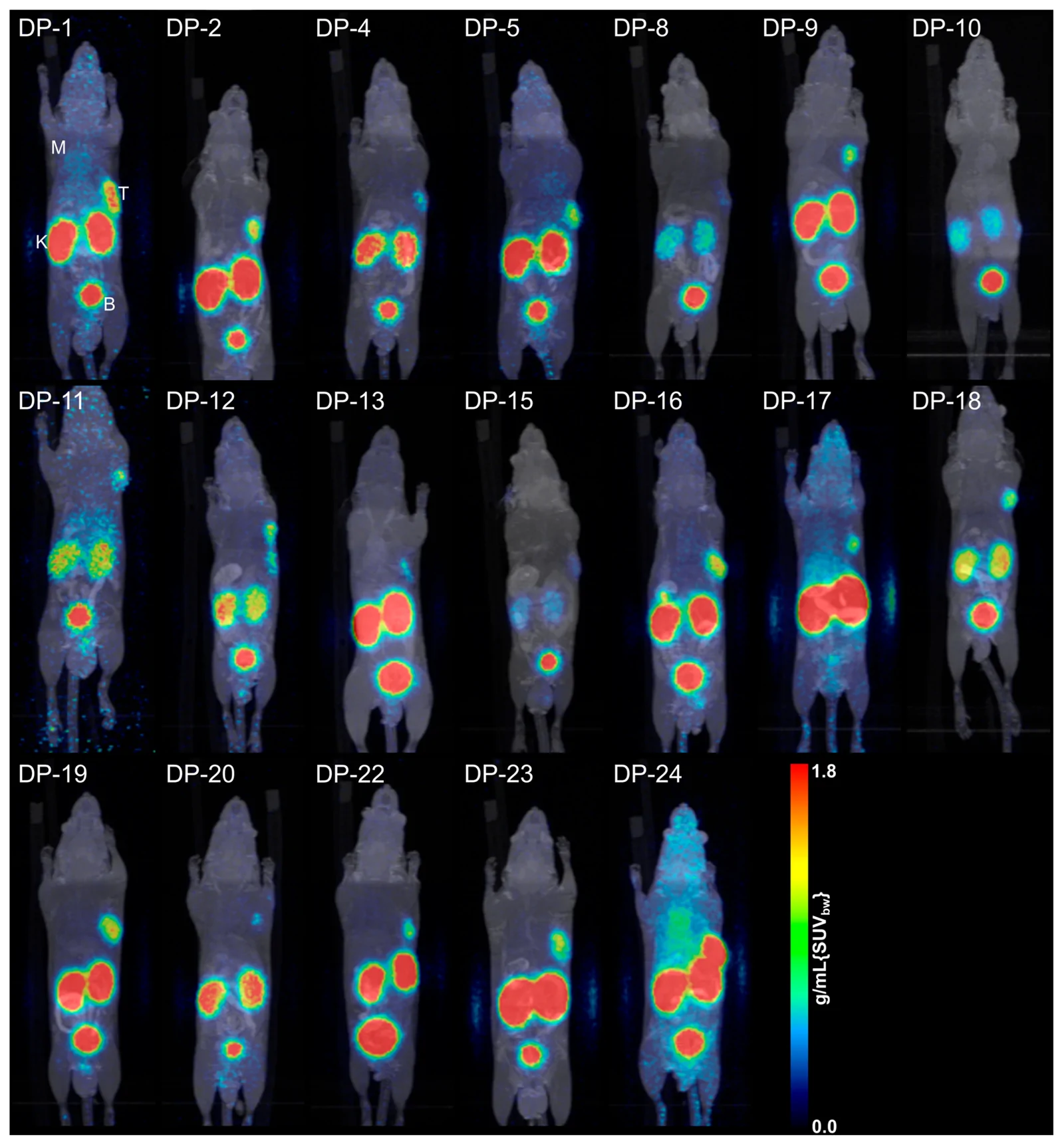
PET/MR maximum intensity projection (MIP) images of LNCaP tumor-bearing mice at 2 h p.i. of 500 pmol of the respective ^68^Ga-labeled compound. DP-1 (DP-Atto590), DP-2 (DP-Alexa594), DP-4 (DP-STAR635P), DP-5 (DP-STAR RED), DP-8 (DP-CF680R), DP-9 (DP-580CP-Methoxy), DP-10 (DP-610CP), DP-11 (DP-Sir-Methyl), DP-12 (DP-SulfoCy5), DP-13 (DP-IRDye800CW), DP-15 (DP-Alexa Fluor 647), DP-16 (DP-SulfoCy7), DP-17 (DP-SulfoCy7.5), DP-18 (DP-Tide Fluor 5WS), DP-19 (DP-Atto490LS), DP-20 (DP-DY-521XL), DP-22 (DP-DY-647), DP-23 (DP-DY-540XL), DP-24 (DP-mFluor Blue 660 SE). Anatomical structures are indicated for DP-1 as follows: T = tumor, K = kidneys, B = bladder, M = muscle. Color scale represents standardized uptake values normalized to body weight (SUVbw).

Static PET imaging at 2 h p.i. revealed substantial variability in tumor and kidney uptake across the compound library (Figure 2), while the overall pattern of organ distribution remained consistent: all compounds predominantly localized to tumors and kidneys, with minimal off-target accumulation. Notably, while the qualitative distribution pattern was conserved across the entire library, with uptake confined predominantly to tumors and kidneys, the magnitude of both tumor and kidney accumulation varied substantially with the conjugated dye. Because the targeting scaffold is identical across all conjugates, this variation is attributable to the dye moiety rather than to the PSMA-binding vector, directly illustrating the impact of dye conjugation on in vivo pharmacokinetics. DP-12 (DP-SulfoCy5) and DP-13 (DP-IRDye800CW) were included as reference compounds, representing two of the most commonly used fluorophores in the field of dual-labeled compounds. DP-12 demonstrated high tumor uptake (SUV_bw_ = 0.60) and low kidney uptake at 2 h p.i. (SUV_bw_ = 0.94). In contrast, DP-13 exhibited lower tumor uptake (SUV_bw_ = 0.26) and, consistent with the dynamic imaging data described below, prolonged kidney retention (SUV_bw_ = 3.25 at 2 h p.i.). Two additional compounds were DP-15 and DP-18, with DP-15 (DP-Alexa Fluor 647; SUV_bw_ = 0.14 tumor, 0.24 kidney) showing lower absolute tumor uptake than DP-12 but favorable rapid kidney washout, and DP-18 (DP-Tide Fluor 5WS; SUV_bw_ = 0.56 tumor, 0.96 kidney) displaying a profile closely comparable to DP-12. Furthermore, DP-5 (DP-STAR RED), DP-9 (DP-580CP-Methoxy), and DP-19 (DP-Atto490LS) exhibited favorable kinetic profiles. DP-10 (DP-610CP) showed lower tumor accumulation for clinical consideration but was notable for its rapid kidney washout, which further supports the favorable pharmacokinetic behavior of the sarcosine-based scaffold. A notable outlier was DP-24 (DP-mFluor Blue 660 SE), which exhibited prolonged blood circulation beyond 2 h p.i., resulting in exceptionally high tumor accumulation (SUV_bw_ = 1.97) and sustained kidney retention (SUV_bw_ = 4.56). In contrast, DP-4 (DP-STAR635P), DP-8 (DP-CF680R), and DP-20 (DP-DY-521XL) showed consistently low tumor uptake in both PET imaging and ex vivo organ distribution (<3.5%ID/g), falling below the range useful for clinical translation. DP-11 (DP-Sir-Methyl), while showing moderate tumor uptake on PET, also fell below this threshold in ex vivo analysis (1.24%ID/g). Dynamic 1 h PET/MR images for all compounds are provided in Supplementary Figure S5. Organ distribution values at 2 h p.i. for all compounds are summarized in Table 3. No significant paravenous injection was observed in any animal; tail uptake remained below 1%ID/g across all compounds.

**Figure 3:**
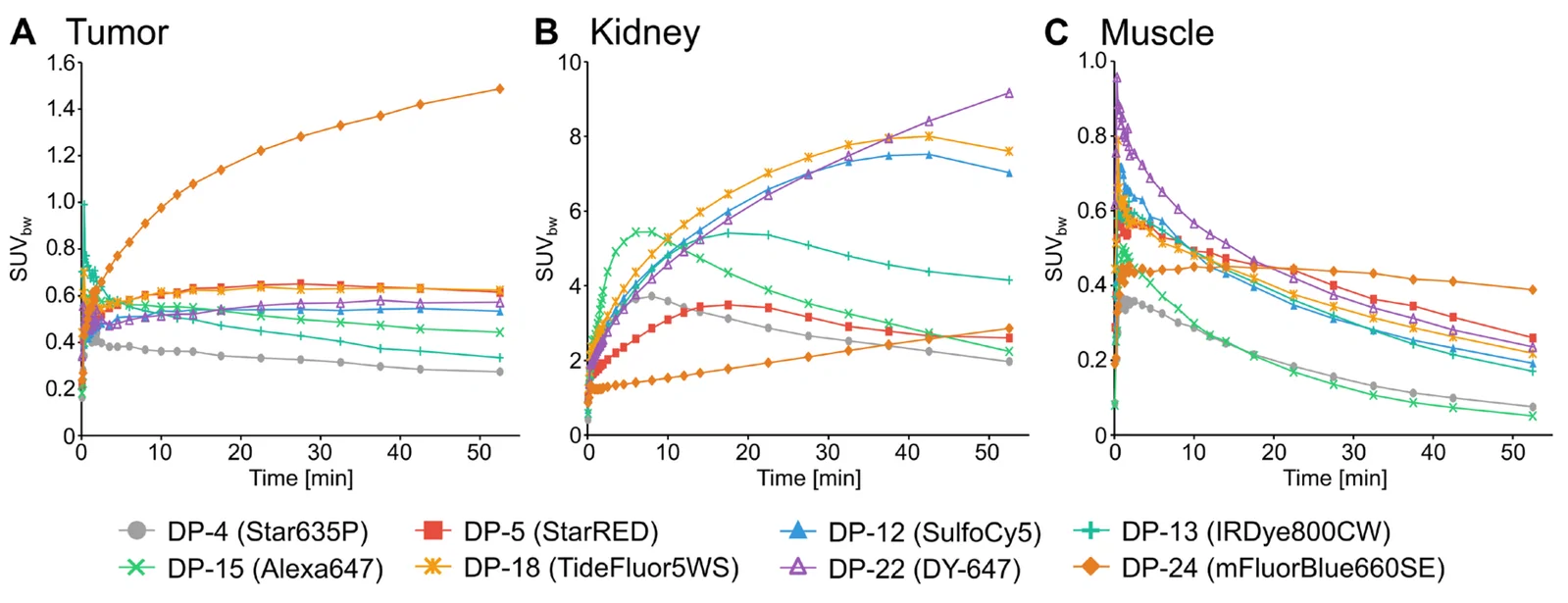
Time–activity curves derived from dynamic PET imaging over 60 min p.i. following administration of 500 pmol ^68^Ga-labeled compound in LNCaP tumor-bearing mice. Tumor (**A**), kidney (**B**), and muscle (**C**) uptake are shown for the seven compounds subsequently evaluated by fluorescence imaging—DP-4 (DP-STAR635P), DP-5 (DP-STAR RED), DP-12 (DP-SulfoCy5), DP-13 (DP-IRDye800CW), DP-15 (DP-Alexa Fluor 647), DP-18 (DP-Tide Fluor 5WS), and DP-22 (DP-DY-647)—and DP-24 (DP-mFluor Blue 660 SE) as a pharmacokinetic outlier. Values are expressed as standardized uptake values normalized to body weight (SUVbw).

To illustrate the diversity in distribution kinetics across the library, time–activity curves (TACs) derived from the 60 min dynamic PET acquisition are shown in Figure 3 for the seven compounds subsequently evaluated by fluorescence imaging—DP-4 (DP-STAR635P), DP-5 (DP-STAR RED), DP-12 (DP-SulfoCy5), DP-13 (DP-IRDye800CW), DP-15 (DP-Alexa Fluor 647), DP-18 (DP-Tide Fluor 5WS), and DP-22 (DP-DY-647)—together with DP-24 (DP-mFluor Blue 660 SE) as a pharmacokinetic outlier. TACs for all 19 compounds are provided in Supplementary Figure S6A,B. For most compounds, tumor uptake reached a plateau within the first 30 min p.i. and remained stable throughout the 60 min acquisition period; in contrast, DP-24 displayed continuously rising tumor uptake over the entire dynamic scan (Figure 3A, Tumor). Kidney kinetics varied substantially across the library: some compounds showed rapid early peaks followed by decline (e.g., DP-8, DP-10, DP-11, DP-15), while others exhibited continuously rising uptake throughout the dynamic scan (e.g., DP-9, DP-16, DP-22, DP-23, DP-24) (Figure 3B, Kidney). Muscle uptake peaked within the first 10 min p.i. and declined thereafter for nearly all compounds, reflecting rapid blood pool washout (Figure 3C, Muscle), whereas DP-24 again exhibited slower kinetics. No relevant accumulation beyond blood pool was observed in non-target organs such as liver or spleen for any compound. The most variable parameters across the library were blood pool circulation time, tumor accumulation, and kidney uptake.

At 2 h p.i., tumor-to-muscle (T/M) ratios varied substantially, with DP-15 (DP-Alexa Fluor 647) exhibiting the highest ratio (38.6), followed by DP-18 (DP-Tide Fluor 5WS; 21.7), DP-12 (DP-SulfoCy5; 9.5), DP-13 (DP-IRDye800CW; 6.5), and DP-24 (DP-mFluor Blue 660 SE; 7.8). Calculated T/M ratios for all compounds are shown in Supplementary Figure S7. Quantitative organ distribution data (Table 3) obtained at 2 h p.i. corroborated the SUV_bw_ values derived from PET imaging.

#### 2.4.2. Fluorescent Imaging

Fluorescent signals were successfully detected ex vivo for all compounds, with absorbance maxima corresponding to either the 700 nm or 800 nm channel. However, large Stokes shift dyes (DP-19: DP-Atto490LS, DP-20: DP-DY-521XL, DP-23: DP-DY-540XL, and DP-24: DP-mFluor Blue 660 SE) could not be assessed with this system, as their excitation maxima fall outside the available wavelengths. Furthermore, direct quantitative comparison of fluorescence intensities between different compounds was not feasible, as each dye exhibits distinct absorbance and emission properties, precluding meaningful cross-compound comparisons. Complete ex vivo fluorescence imaging data for all compounds are provided in Supplementary Figure S8.

**Figure 4:**
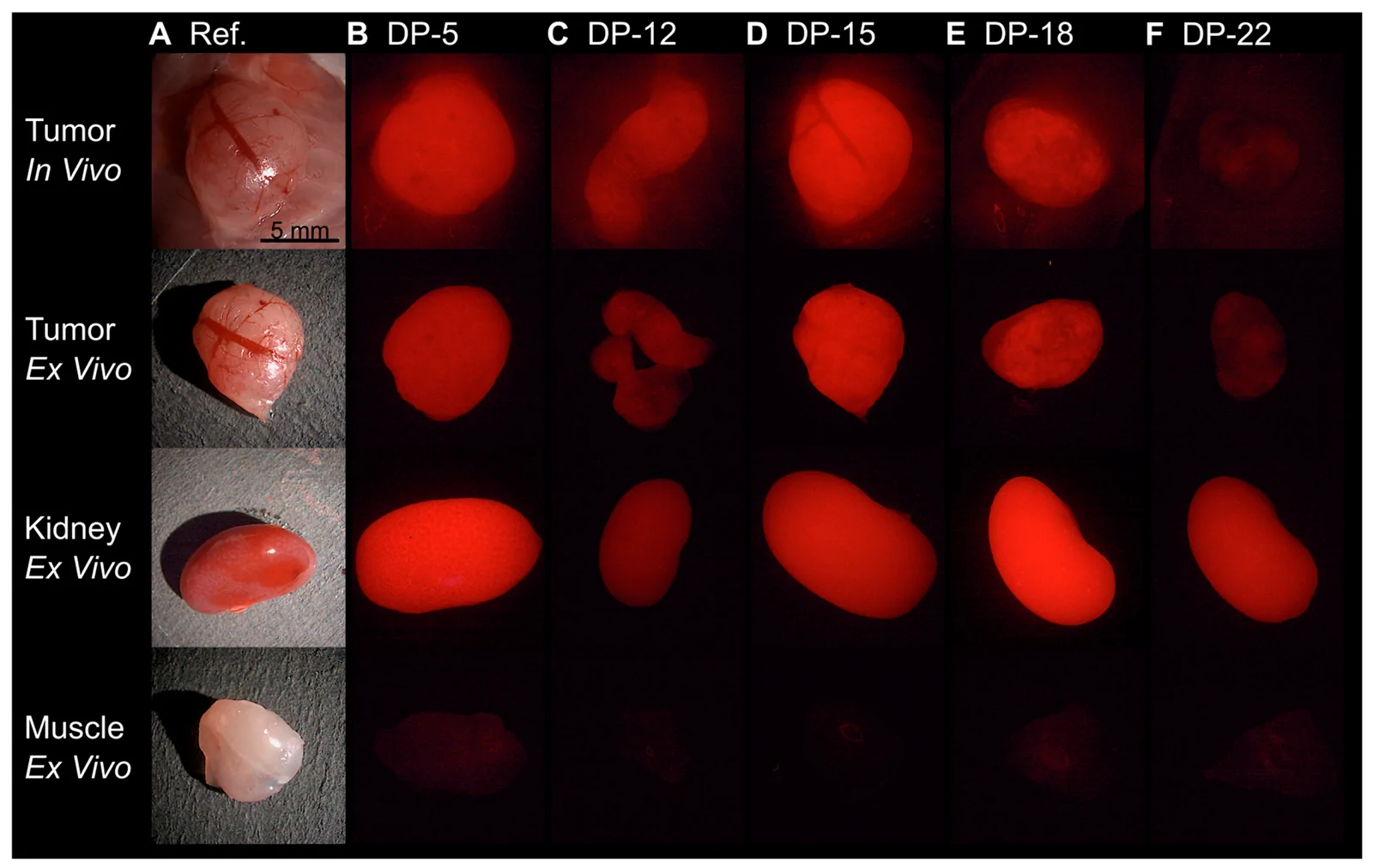
Representative white light (**A**) and corresponding fluorescence images (excitation 637 nm; fluorescence signal shown in red) of (**B**) DP-5 (DP-STAR RED), (**C**) DP-12 (DP-SulfoCy5), (**D**) DP-15 (DP-Alexa Fluor 647), (**E**) DP-18 (DP-Tide Fluor 5WS), and (**F**) DP-22 (DP-DY-647). For each compound, images show tumor in vivo (with skin opened), tumor ex vivo, kidney ex vivo and muscle ex vivo. Scale bar, 5 mm. All panels share the same scale.

In vivo fluorescence imaging was performed using a handheld spectroscopic endoscope system (Henke-Sass, Wolf GmbH). The system operates at a fixed excitation wavelength and emission filter, which was specifically designed to match the absorbance spectral properties of SulfoCy5 (DP-12). Consequently, fluorescent signals could only be reliably detected for compounds bearing dyes with similar characteristics. Nevertheless, all compounds were subjected to in vivo imaging to assess potential autofluorescence or other sources of false-positive signals. From the compound library, seven conjugates—DP-4 (DP-STAR635P), DP-5 (DP-STAR RED), DP-11 (DP-Sir-Methyl), DP-12 (DP-SulfoCy5), DP-15 (DP-Alexa Fluor 647), DP-18 (DP-Tide Fluor 5WS), and DP-22 (DP-DY-647)—exhibited fluorescent properties compatible with the endoscope detection range. Figure 4 shows the fluorescent signal in red from the tumor detected with skin removed, and after tumor excision, alongside an isolated kidney as a positive reference.

**Figure 5:**
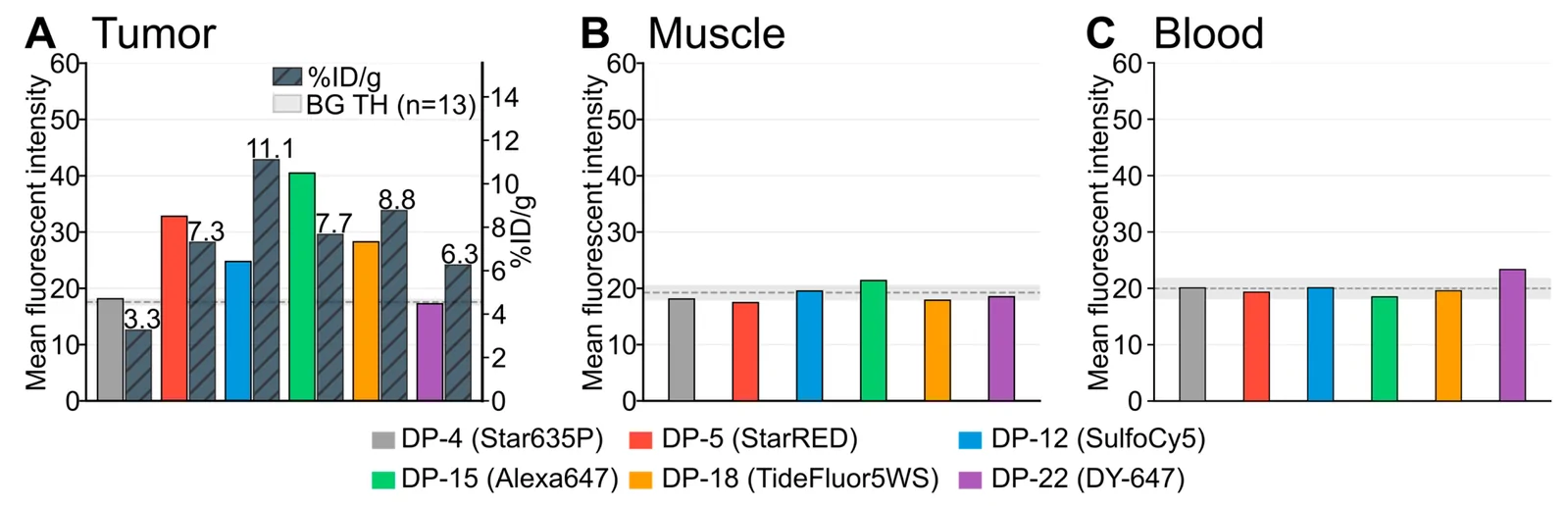
Quantitative fluorescence image analysis of DP-4 (DP-STAR635P), DP-5 (DP-STAR RED), DP-12 (DP-SulfoCy5), DP-15 (DP-Alexa Fluor 647), DP-18 (DP-Tide Fluor 5WS), and DP-22 (DP-DY-647). Tumor (**A**): mean fluorescence intensity in tumor tissue (colored bars, left y-axis) with corresponding ex vivo tumor uptake (%ID/g; hatched bars, right y-axis; values indicated above bars). Muscle (**B**) and Blood (**C**): mean fluorescence intensities in muscle tissue and blood serving as negative controls. The dashed line and gray shaded region indicate the fluorescence background threshold ± SD, calculated from compounds spectrally incompatible with the endoscopic setup (*n* = 13). *n* = 1.

DP-11 (DP-Sir-Methyl) was excluded from further analysis due to its unfavorable biodistribution profile (tumor uptake 1.24%ID/g; Table 3) and the absence of detectable fluorescent signal. DP-4 (DP-STAR635P) was retained in the quantitative analysis (Figure 5) as an informative near-threshold reference despite its lower tumor uptake (3.26%ID/g). DP-5 (DP-STAR RED), DP-12 (DP-SulfoCy5), DP-15 (DP-Alexa Fluor 647), and DP-18 (DP-Tide Fluor 5WS) all displayed strong fluorescent signals with clear contrast against background and surrounding tissue. Interestingly, no fluorescent signal was detected from tumors with DP-22 (DP-DY-647), despite a tumor uptake of 6.26%ID/g (Figure 4 and Figure 5). Kidney tissue showed detectable fluorescent signals for DP-22, comparable to the other compounds, indicating that the dye itself was functional. Muscle tissue showed no fluorescent signal for any compound. Quantitative analysis of fluorescence images revealed that DP-5 (DP-STAR RED), DP-12 (DP-SulfoCy5), DP-15 (DP-Alexa Fluor 647), and DP-18 (DP-Tide Fluor 5WS) exhibited clearly distinguishable mean tumor fluorescent signals above the background threshold defined by spectrally incompatible compounds (*n* = 13; Figure 5A, Tumor). DP-4 (DP-STAR635P) and DP-22 (DP-DY-647) remained within the threshold region, consistent with the absence of a detectable tumor signal in the corresponding endoscopic images (Figure 4F). Muscle fluorescence remained at background level across all compounds (Figure 5B, Muscle), confirming the absence of a non-specific tissue signal. As expected, the fluorescence intensity rankings did not correspond to the rankings of ex vivo tumor uptake (%ID/g, overlaid in Figure 5A, Tumor): although DP-15 and DP-12 showed the highest fluorescence signals, their ex vivo tumor uptake values were intermediate within the evaluated subset, while DP-22, which had substantial ex vivo tumor uptake (6.26%ID/g), produced only background-level fluorescence.

**Figure 6:**
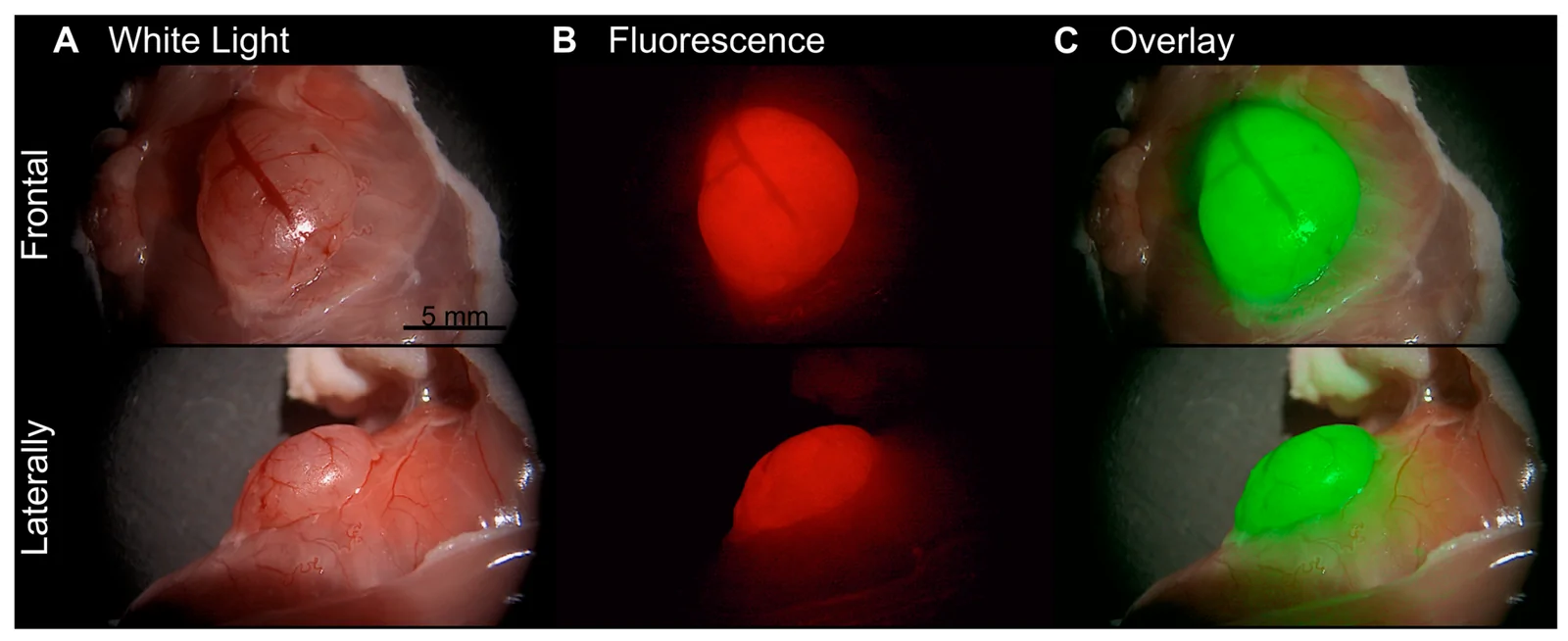
Representative imaging of DP-15 (DP-Alexa Fluor 647) tumor visualization. (**A**) White light image of the tumor (skin removed). (**B**) Fluorescence feedback (red) measured at 637 nm excitation. (**C**) Overlay of thresholded fluorescence intensity data applied as a color mask onto the white light image, enabling visualization of spatial fluorescent signal distribution in relation to anatomical structures. Scale bar, 5 mm. All panels share the same scale.

As a translational demonstration of potential real-world clinical applications, we generated a fluorescence overlay to visualize compound localization. Thresholded fluorescence intensity data were extracted and used to generate a color mask, which was subsequently overlaid onto the corresponding white light image of the tumor with skin removed. This composite representation enables direct spatial correlation between fluorescent signal and anatomical tissue structures, illustrating how fluorescence-guided imaging could assist in intraoperative tumor delineation. A representative example using DP-15 (DP-Alexa Fluor 647) is shown in Figure 6.

#### 2.4.3. Organ Distribution

**Figure 7:**
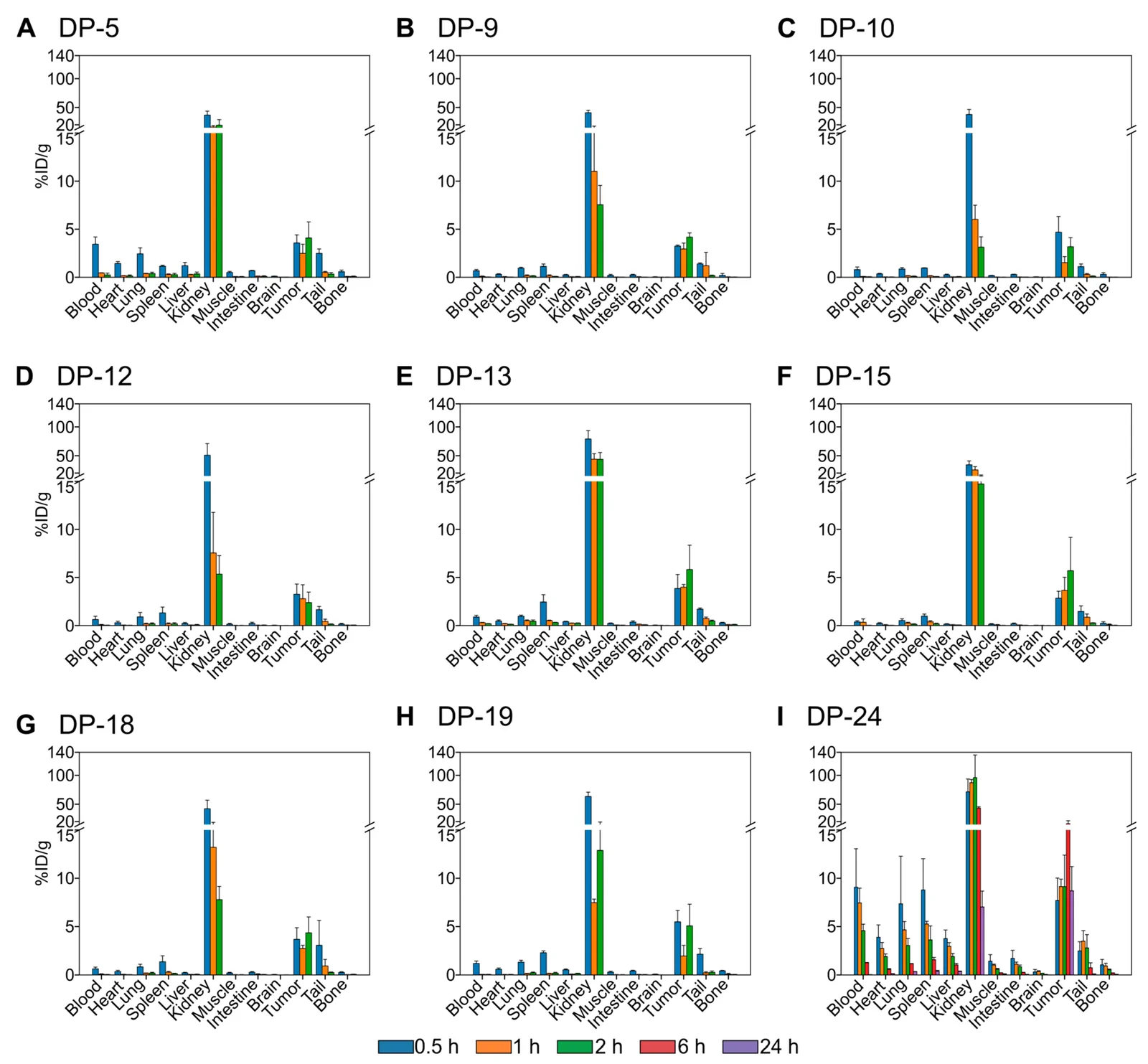
Organ distribution of selected ^177^Lu-labeled compounds expressed as %ID/g. (**A**) DP-5 (DP-STAR RED), (**B**) DP-9 (DP-580CP-Methoxy), (**C**) DP-10 (DP-610CP), (**D**) DP-12 (DP-SulfoCy5), (**E**) DP-13 (DP-IRDye800CW), (**F**) DP-15 (DP-Alexa Fluor 647), (**G**) DP-18 (DP-Tide Fluor 5WS), (**H**) DP-19 (DP-Atto490LS), and (**I**) DP-24 (DP-mFluor Blue 660 SE). Compounds were administered at 60 pmol and organs were collected at 0.5, 1, and 2 h p.i.; DP-24 was additionally evaluated at 6 and 24 h p.i. due to its prolonged circulation time. Data represent mean ± SD (*n* = 3 per time point).

The organ distribution data summarized in Figure 7 demonstrate that all compounds, with the exception of DP-24 (DP-mFluor Blue 660 SE), displayed favorable biodistribution profiles, with off-target organ uptakes below 1%ID/g at 2 h p.i. In contrast, tumor uptake and kidney retention showed substantial variability across the compound library, ranging from 1.24%ID/g to 22.94%ID/g in tumors and 4.71%ID/g to 81.94%ID/g in kidneys, respectively. Given its markedly different distribution pattern, DP-24 will be evaluated separately. Compound selection for more detailed biodistribution studies was guided by the evaluation framework defined in the Results opener: compounds demonstrating either favorable PET-derived pharmacokinetics (moderate-to-high tumor uptake with decreasing kidney retention at 1–2 h p.i.) or successful fluorescence detection with the endoscopic setup were prioritized. Based on this rationale, DP-5 (DP-STAR RED), DP-9 (DP-580CP-Methoxy), DP-10 (DP-610CP), DP-12 (DP-SulfoCy5), DP-13 (DP-IRDye800CW), DP-15 (DP-Alexa Fluor 647), DP-18 (DP-Tide Fluor 5WS), DP-19 (DP-Atto490LS), and DP-24 (DP-mFluor Blue 660 SE) were selected for detailed biodistribution analysis, as shown in Figure 7, with DP-12 and DP-13 used as reference compounds.

The general organ distribution patterns observed after PET/MR imaging (Table 3) were confirmed in the dedicated biodistribution study for the selected compounds. Across all evaluated compounds, tumor %ID/g was lower at the reduced peptide dose (60 pmol, ^177^Lu) than at the imaging dose (500 pmol, ^68^Ga). The differences could reflect the effects of the administered experimental protocol, including anesthesia or mass dependence of PSMA-targeted uptake. Consistent with the imaging data, no accumulation in off-target organs was observed at any time point for any compound. Importantly, tumor accumulation remained stable over time and did not show washout. In contrast, distinct differences in renal elimination kinetics were observed across the compound library. Three patterns emerged: first, DP-9 (DP-580CP-Methoxy), DP-10 (DP-610CP), DP-12 (DP-SulfoCy5), DP-18 (DP-Tide Fluor 5WS), and DP-19 (DP-Atto490LS) (Figure 7B–D,G,H) demonstrated rapid renal washout within the first 30 to 60 min p.i. Second, DP-5 (DP-STAR RED) and DP-15 (DP-Alexa Fluor 647) (Figure 7A,F) exhibited moderate kidney accumulation combined with prolonged retention. Third, DP-13 (DP-IRDye800CW) and DP-24 (DP-mFluor Blue 660 SE) (Figure 7E,I) displayed high kidney uptake with extended retention times, suggesting strong renal binding or slower elimination kinetics. Notably, extended evaluation of DP-24 revealed substantial renal washout by 24 h p.i. (7.03 ± 1.65%ID/g), while tumor uptake remained high (8.70 ± 2.51%ID/g).

#### 2.4.4. PC-3 Negative Control

To verify PSMA specificity, DP-13 (DP-IRDye800CW) was additionally evaluated in mice bearing PSMA-negative PC-3 xenograft. No PSMA-specific tumor accumulation was observed by PET/MR imaging or organ distribution analysis, while renal kinetics remained comparable to those observed in LNCaP tumor-bearing animals [Supplementary Figure S9].

## 3. Discussion

The field of dual-labeled compounds for FGS would benefit from a broad library of fluorescent dyes suitable for specific applications and clinical needs. Since conjugation of fluorescent dyes—contributing 25–50% of the molecular weight of small peptide binders—can significantly affect overall compound behavior, detailed chemical characterization and both in vitro and in vivo analyses are essential for preclinical evaluation. Several dual-labeled PSMA-targeting compounds have been reported to date, including PSMA-11-derived dye conjugates bearing IRDye800CW [15], the PSMA-617-based compound bearing fluorescein (PSMA-921-926) or IRDye800CW (PSMA-927) [11], and the more recently described DPR1008 [17]. Several dual-labeled PSMA tracers have also been evaluated in clinical settings, including PSMA-914, a PSMA-11-derived IRDye800CW conjugate used for combined preoperative PSMA-PET/CT and intraoperative fluorescence imaging during radical prostatectomy [18], as well as PSMA-HSG, a SulfoCy5-bearing hybrid tracer derived from the PSMA-I&S/I&T family, recently evaluated in a first-in-human study demonstrating feasibility of combined radio- and fluorescence-guided surgery [19]. However, these efforts have predominantly focused on individual NIR fluorophores or single dye–scaffold combinations, and systematic comparisons across structurally diverse dye classes on a uniform scaffold remain limited. Building upon our earlier work with PSMA-927, the present study expands the scope from a single NIR dye to a comprehensive library of 19 fluorescent dyes spanning NIR, large Stokes shift, and STED dyes, all conjugated to a linker-optimized PSMA-617-derived scaffold yielding favorable pharmacokinetic characteristics.

Comparing our in vitro data with clinically established compounds such as PSMA-617 [20,21] and PSMA-11 [9], as well as the aforementioned dual-labeled agents, we observed that dye conjugation tends to decrease specific binding affinities as well as surface-binding and internalization rates. Nevertheless, binding affinities remained in the low nanomolar range (Ki = 18–87 nM, from 13.2 nM precursor), and internalization could still be observed (0.09–0.79%AA/105 cells, from 2.2%AA/105 cells precursor), while all compounds retained strongly hydrophilic characteristics (logD = −3.72 to −1.79). Serum stability appeared most susceptible to dye-induced effects: while most compounds showed favorable stability in human serum (94–100% intact after 24 h), pronounced species-dependent degradation was observed for DP-4 (DP-STAR635P), DP-11 (DP-Sir-Methyl), DP-13 (DP-IRDye800CW) and DP-17 (DP-SulfoCy7.5) in murine serum.

The most pronounced effects of fluorescent dye conjugation were observed in vivo. Dynamic PET imaging revealed that the majority of compounds reached peak tumor uptake within the first 30 min p.i., with kidney retention decreasing substantially, though to varying degrees across the library, by 2 h p.i. (Figure 2 and Figure 3), suggesting that the sarcosine spacer backbone provides favorable pharmacokinetic properties for same-day surgical workflows. The advantage of the sarcosine-based scaffold becomes evident when comparing DP-13 (DP-IRDye800CW) with the structurally related PSMA-927 [10], which employs the same NIR dye but without a sarcosine spacer: blood clearance was accelerated and off-target organ accumulation was reduced, while comparable tumor uptake was maintained. The consequences of omitting such a spacer become even more pronounced in DPR1008, which requires pre-administration 12–24 h before surgical intervention [17], and in PSMA-HSG (bearing SulfoCy5 on the PSMA-I&S scaffold), which exhibits prolonged blood pool circulation and substantial off-target accumulation [19]. These observations collectively highlight the advantage of the sarcosine-based spacer architecture for reducing background signal. Importantly, the PSMA-specificity of tumor accumulation was confirmed by the absence of detectable uptake in PC-3 (PSMA-negative) xenografts for DP-13. The absence of accumulation in PSMA-negative PC-3 tumors, despite preserved renal kinetics, argues against a substantial enhanced permeability and retention (EPR)-mediated non-specific contribution to tumor uptake for these dye conjugates.

Fluorescence imaging using a clinical-grade handheld endoscopic system revealed an apparent dissociation between radiotracer-derived tumor uptake (%ID/g) and fluorescence signal intensity (Figure 5). Due to the fixed excitation wavelength optimized for SulfoCy5, seven compounds were spectrally compatible with the endoscope (Figure 4), of which six produced sufficient tumor signal for quantitative analysis (Figure 5). Notably, structurally homologous dyes DP-4 (DP-STAR635P) and DP-5 (DP-STAR RED) exhibited markedly different tumor imaging outcomes, likely reflecting a threshold effect related to tumor accumulation. Similarly, DP-22 (DP-DY-647) showed strong kidney fluorescence yet no tumor signal despite appreciable uptake (6.26%ID/g). One possible explanation, which remains to be tested, is that microenvironmental factors, for example, local pH [22], protein interactions [23], or tissue-specific quenching [24], modulate fluorophore performance independently of absolute tracer accumulation. The present data cannot discriminate among these candidate mechanisms; resolving the basis of the DP-22 signal loss would require dedicated photophysical characterization in the relevant tissue context, which we identify as a direction for future work. These findings underscore that dye-inherent optical characteristics must be weighted equally alongside pharmacokinetic parameters when selecting fluorophores for FGS. Nevertheless, when a suitable candidate is identified, the clinical benefit is substantial, as illustrated by precise tumor delineation under fluorescence overlay (Figure 6).

Evaluation of the pharmacokinetic profiles across the library revealed no single physicochemical determinant of biodistribution, as renal retention did not track in a consistent way with net charge, lipophilicity or molecular weight. However, NIR dyes (DP-IRDye800CW, DP-SulfoCy7, DP-SulfoCy7.5) consistently exhibited slower distribution kinetics and prolonged renal retention, while structurally related visible-range dyes, such as DP-12 (DP-SulfoCy5), DP-15 (DP-Alexa Fluor 647), and DP-22 (DP-DY-647), displayed markedly divergent pharmacokinetic profiles, suggesting that subtle structural modifications critically influence in vivo behavior. Several compounds, including DP-9 (DP-580CP-Methoxy), DP-10 (DP-610CP), DP-12 (DP-SulfoCy5), DP-18 (DP-Tide Fluor 5WS), and DP-19 (DP-Atto490LS), exhibited rapid renal washout with near-complete elimination within 1 h p.i. while maintaining high tumor retention (Figure 7). This fast-eliminating profile aligns with the same-day surgical workflow targeted by the present evaluation, enabling preoperative PET imaging and intraoperative fluorescence guidance within a single clinical session without requiring pre-administration the day before surgery. The higher T/M ratios observed for fast-eliminating visible-range dyes (DP-15: 38.6; DP-18: 21.7; DP-12: 9.5) compared to the NIR fluorophore DP-13 (6.5) reflect more rapid clearance from blood rather than differences in absolute tumor uptake.

DP-24 (DP-mFluor Blue 660 SE) warrants separate consideration. While its prolonged blood pool residence and slow distribution kinetics fall outside the same-day workflow adopted here, DP-24 produced the highest absolute tumor uptake observed across the library (22.94%ID/g at 2 h p.i.), with stable retention at later timepoints and substantially decreased kidney activity by 24 h p.i. These observations suggest that DP-24 may be better suited to a later imaging procedure, analogous to established ICG-based FGS protocols, in which the tracer is administered 12–24 h before surgery, rather than to the same-day workflow targeted in the present study.

Current clinical robotic surgical platforms, including the da Vinci system (Intuitive Surgical), Hugo RAS (Medtronic), and Versius (CMR Surgical), are equipped exclusively with NIR fluorescence imaging systems optimized for ICG detection (~800 nm) [25], which has driven the predominant use of NIR fluorophores. However, our data demonstrate that NIR dyes are associated with unfavorable biodistribution profiles, whereas visible-range dyes exhibited rapid pharmacokinetics without substantial limitation from tissue autofluorescence. The faster elimination kinetics could additionally permit higher administered doses, potentially compensating for reduced tissue penetration. Beyond pharmacokinetic considerations, large Stokes shift dyes such as DP-19 (DP-Atto490LS; 168 nm spectral separation) may offer optical advantages through more stringent excitation light filtering, reducing excitation backscatter and bleed-through into the emission channel, thereby improving signal-to-background ratio, warranting further investigation.

Taken together, this work advances the field in three respects. First, by evaluating 19 structurally diverse dyes on a single uniform scaffold, it isolates dye-dependent pharmacokinetic and optical effects from scaffold-dependent ones, which single-dye studies cannot resolve. Second, it establishes that fluorescence signal and radiotracer-derived uptake are dissociated, so photophysical and pharmacokinetic suitability must be evaluated as independent selection criteria. Third, it demonstrates that fast-clearing visible-range dyes can achieve tumor-to-background contrast competitive with or exceeding established NIR fluorophores within a same-day surgical workflow. These findings define concrete next steps: dose optimization of the identified leads, validation in orthotopic and transgenic models, and development of broader-spectrum endoscopic hardware to exploit the large-Stokes-shift candidates that the current SulfoCy5-optimized system cannot detect.

Two separate levels of evaluation should be distinguished when interpreting these results. Intrinsic pharmacokinetic performance, derived from PET/MR imaging and biodistribution, is independent of the fluorescence detection hardware and was assessed across the entire library. Clinical-candidate selection, in contrast, is constrained by the detection window of the available endoscopic system, which is optimized for the SulfoCy5 range. As a consequence, the identification of DP-12, DP-15 and DP-18 as leads reflects a combination of favorable pharmacokinetics and compatibility with current hardware, rather than intrinsic optical superiority over the remainder of the library. Several compounds with favorable pharmacokinetic profiles, in particular, the large Stokes shift dyes, could not be ranked as clinical leads simply because their excitation falls outside the accessible range of the present endoscope. The ranking of clinical candidates would therefore be expected to shift with broader-spectrum imaging hardware, which is the reason spectrally incompatible compounds were retained throughout the in vivo characterization rather than excluded from the outset.

Several limitations of the present study should be acknowledged as well. First, the evaluation was performed under standardized tracer-level conditions in order to isolate dye-dependent effects across the library. Absolute fluorescence brightness at the higher mass doses used in clinical FGS, where receptor saturation and concentration-dependent self-quenching may modulate signals, was not assessed and remains to be established for the lead candidates as part of dose optimization. Second, subcutaneous LNCaP xenografts do not reproduce orthotopic prostatic architecture or adjacent critical structures and are subject to EPR effects, which have been shown to contribute to non-specific tracer accumulation in this model [26]. Validation of lead candidates in orthotopic or transgenic PSMA-expressing models is therefore an important next step. Third, biodistribution was characterized over the 0.5–2 h same-day surgical window targeted here. Residual kidney and urinary activity at later timepoints, and the potential for urinary signals to interfere with a pelvic surgical field, were not assessed and warrant dedicated evaluation prior to clinical implementation. Fourth, the visible-range fluorophores that emerged as leads offer shallower tissue penetration than NIR dyes. Visible-light fluorescence imaging is generally limited to few mm of tissue, compared with the millimeter to centimeter range accessible in the near-infrared window [27], which restricts visible-range candidates to surface-exposed or surgically opened fields. This is an inherent optical constraint independent of the pharmacokinetic advantages reported here. Finally, the present work did not include direct intraoperative surgical validation, which, together with the considerations above, defines the principal objectives of subsequent translational studies.

Future studies should evaluate long-term photostability under surgical conditions and assess the identified lead candidates in first-in-human trials.

## 4. Materials and Methods

### 4.1. Chemical Synthesis & Characterization

#### 4.1.1. Fluorescent Dye Library

The dye library (Table 1) was selected to cover the far-red visible to near-infrared (NIR) spectral range, with emphasis on three distinct dye categories: (1) three NIR-emitting dyes suitable for deep tissue imaging (DP-13, DP-16, DP-17); (2) four large Stokes shift dyes to assess whether reduced spectral overlap between excitation and emission improves imaging contrast (DP-19, DP-20, DP-23, DP-24); and (3) twelve STED-compatible dyes, specifically developed for superior brightness and photostability, to evaluate their potential for enhanced intraoperative visualization. STED compatibility was used here solely as a selection criterion for high brightness and photostability. Stimulated emission depletion itself is a microscopy modality and was not, and cannot be, assessed with the macroscopic imaging systems used in this study.

#### 4.1.2. Synthesis of Fluorescent Dye Conjugates

The peptide was dissolved in phosphate-buffered saline (PBS, pH 8.3, 1 M). The respective dye was dissolved in anhydrous, amine-free DMSO and added at 20% molar excess relative to the peptide. The reaction mixture was incubated overnight at room temperature (RT) under gentle agitation, protected from light.

Purification was performed by semi-preparative reversed-phase high-performance liquid chromatography (RP-HPLC) (Agilent 1260 series, Agilent Technologies, Inc., CA, USA) through a Chromolith^®^ SemiPrep RP-18e column (100 × 10 mm, Merck KGaA, Darmstadt, Germany). Mobile phases consisted of water with 0.1% trifluoroacetic acid (TFA) and acetonitrile with 0.1% TFA. Product-containing fractions were identified by matrix-assisted laser desorption/ionization time-of-flight mass spectrometry (MALDI-TOF-MS, Microflex; Bruker, MA, USA) and subsequently pooled.

Pooled fractions were snap-frozen in liquid nitrogen and lyophilized overnight. Final products were dissolved in DMSO to prepare 10 mM and 1 mM stock solutions. Purity was confirmed by analytical RP-HPLC using a Chromolith Performance RP-18e column (100 × 4.6 mm, Merck KGaA, Darmstadt, Germany), with a threshold of >95% for all conjugates used in subsequent experiments.

#### 4.1.3. Radiolabeling with ^68^Ga and ^177^Lu

For ^68^Ga-labeling, 40 µL HEPES buffer (1 M, pH 4.1, containing 2.5 µg/µL ascorbic acid) was mixed with 50–70 MBq ^68^Ga (Gallia Pharm generator; Eckert & Ziegler, Berlin, Germany) in 40 µL strong cation exchange (SCX) solution. Subsequently, 3 µL of the respective compound (1 mM in DMSO; 3 nmol) was added, and the reaction mixture was incubated at 95 °C for 10 min. For ^177^Lu-labeling, 50 µL ammonium acetate buffer (0.5 M, pH 5.4, containing 2 µg/mL ascorbic acid) was mixed with 30 MBq ^177^Lu (DSD Pharma GmbH, Purkersdorf, Austria) in 10 µL 0.04 M HCl. A volume of 3 µL of the respective compound was added (1 mM in DMSO; 3 nmol) and the mixture was incubated at 95 °C for 10 min.

Radiochemical purity was assessed by radio-HPLC and radio-TLC, with a radiochemical purity of >99% required for subsequent experiments. Radio-HPLC analysis was performed on a Latek (P-402; Latek Labortechnik & Analysen GmbH, Eppelheim, Germany) system equipped with a FlowStar^2^ LB-514 radio detector (FlowStar^2^ LB-514; Berthold Technologies GmbH & Co. KG, Bad Wildbad, Germany)and a Chromolith Performance RP-18e column (100 × 4.6 mm), using a gradient from 90% water/0.1% TFA to 90% acetonitrile/0.1% TFA over 6 min. Radio-TLC was performed on a miniGITA Elysia Raytest (Elysia-raytest GmbH, Straubenhardt, Germany) system using silica gel strips (1 × 8 cm). Mobile phases consisted of DMF/10% ammonium acetate buffer (1:1, *v*/*v*) for ^68^Ga-labeled compounds and acetonitrile/water (1:1, *v*/*v*) for ^177^Lu-labeled compounds.

#### 4.1.4. Lipophilicity (logD) Determination

The ^68^Ga-radiolabeled compound was diluted with PBS (1 M, pH 7.4) to a final concentration of 250 nM. In triplicate, 6 µL of this dilution was added to a mixture of 500 µL octan-1-ol and 494 µL PBS (1 M, pH 7.4). Samples were vortexed thoroughly and centrifuged (810× *g*, 4 °C, 10 min) to achieve phase separation. Two aliquots (100 µL each) were collected from both the organic (upper) and aqueous (lower) phases in randomized order. Radioactivity in each aliquot was measured using a gamma counter (2470 WIZARD_2_^TM^ PerkinElmer Inc, Waltham, MA, USA), and the distribution coefficient was calculated as logD = log (mean counts in octanol phase/mean counts in aqueous phase). Experiments were performed in 3 independent replicates.

### 4.2. In Vitro

#### 4.2.1. Serum Stability

The ^177^Lu -radiolabeled compounds were incubated with 100 µL of both murine and human serum at 37 °C. Aliquots were collected at 0, 1, 2, 6, 24, 48 and 72 h post incubation. Serum proteins were precipitated by addition of acetonitrile, followed by centrifugation (13,000× *g*, 4 °C, 5 min). The supernatant was analyzed by radio-HPLC using an Agilent 1200 system (Agilent Technologies, Inc., CA, USA) equipped with an Elysia RAMONA Star radio detector (Elysia-raytest GmbH, Straubenhardt, Germany) and a Chromolith Performance RP-18e column (100 × 4.6 mm). A gradient from 95% water/0.1% TFA to 95% acetonitrile/0.1% TFA over 15 min was applied. Stability was expressed as the percentage of intact compound relative to total radioactivity.

#### 4.2.2. Cell Culture

LNCaP cells (PSMA-positive) were cultured in RPMI 1640 Medium with GlutaMAX^TM^ (Gibco; Thermo Fisher Scientific Inc, Waltham, MA, USA) supplemented with 10% fetal bovine serum, 1% penicillin/streptomycin, and 1% sodium pyruvate at 37 °C in a humidified atmosphere containing 5% CO_2_. For passaging, cells were washed with PBS, detached using trypsin (2.5 mL, 5 min, 37 °C), and resuspended in fresh medium. Cell density was determined using an automated cell counter (Vi-CELL XR; Beckmann Coulter GmbH, Krefeld, Germany). PC-3 cells (PSMA-negative) were cultured under identical conditions as described for LNCaP cells and served as a negative control for in vivo specificity experiments. LNCaP cells (PSMA-positive; RRID:CVCL_0395) and PC-3 cells (PSMA-negative; RRID:CVCL_0035) were authenticated by SNP-based Multiplex Cell Authentication against the cellosaurus cell database (MCA; Multiplexion GmbH, Heidelberg, Germany; both lines 100% identity, most recent authentication June 2026) and routinely tested for mycoplasma contamination.

#### 4.2.3. Internalization Assay

Internalization of ^177^Lu-labeled compounds was assessed in LNCaP cells. Cells were seeded at 1 × 10^5^ cells/well in 24-well plates (Greiner CELLSTAR^®;^; Greiner Bio-One International GmbH, Kremsmünster, Austria) pre-coated with poly-L-lysine (0.01% in PBS, 30 min, RT) and incubated for 24 h at 37 °C in a humidified atmosphere containing 5% CO_2_. The radiolabeled compound was diluted in medium to a final concentration of 30 nM and added to the cells (250 µL/well) after discarding the overnight medium. To determine non-specific binding, parallel wells were co-incubated with an additional 500 µM 2-PMPA as a blocking agent. Cells were incubated for 45 min at either 37 °C or 4 °C. After incubation, cells were washed three times with ice-cold PBS (1 mL). Surface-bound radioactivity was removed by two consecutive incubations with ice-cold glycine–HCl buffer (500 µL, 5 min each), and fractions were pooled. Following an additional wash with ice-cold PBS (1 mL), internalized activity was collected by cell lysis with 0.3 M NaOH (500 µL). All samples were filled up to 1 mL to avoid volume effects. Radioactivity in all fractions was quantified using a gamma counter (2470 WIZARD_2_^TM^, PerkinElmer Inc, Waltham, MA, USA). Internalization was expressed as percentage of applied activity (%AA/10^5^ cells). Assay was performed in 3 independent replicates.

#### 4.2.4. Binding Affinity (IC_50_) Determination

Binding affinities were determined by competitive binding assay in LNCaP cells using [^68^Ga]Ga-PSMA-10 as the reference (K_d_ = 3.8 ± 1.8 nM, [28]). Cells were seeded at 1 × 10^5^ cells/well in 96-well plates (CELLSTAR^®^; Greiner Bio-One International GmbH, Kremsmünster, Austria) pre-coated with poly-L-lysine (0.01% in PBS, 30 min, RT) and incubated overnight at 37 °C in a humidified atmosphere containing 5% CO_2_. Serial dilutions of the cold test compounds were prepared in culture medium containing 0.05% Tween-20 at final concentrations of 0, 0.5, 1, 2.5, 5, 10, 25, 50, 100, 500, 1000, and 5000 nM. The [^68^Ga]Ga-PSMA-10 was diluted to 150 nM in medium. For incubation, 50 µL of the respective cold compound dilution and 3 µL of 150 nM [^68^Ga]Ga-PSMA-10 were added to each well containing 50 µL cells. After incubation for 1 h at 37 °C, cells were placed on ice, washed twice with ice-cold PBS (300 µL), and lysed with 0.3 M NaOH (150 µL). Cell-associated radioactivity was quantified using a gamma counter (2470 WIZARD_2_ PerkinElmer). IC_50_ values were calculated by nonlinear regression analysis using GraphPad Prism (version number 10.4.2; Boston, MA, USA), and inhibition constants (K_i_) were derived using the Cheng–Prusoff equation. Assay was performed in 3 independent replicates.

### 4.3. In Vivo

#### 4.3.1. Ethical Approval

All animal experiments were approved by the *Regierungspräsidium Freiburg* (G23/068) and conducted in accordance with German Animal Welfare Act/EU Directive 2010/63/EU. Animals were housed under standard conditions with a 12 h light/dark cycle and ad libitum access to food and water. Humane endpoints were defined, and animal welfare was monitored daily throughout the study.

#### 4.3.2. PET/MR Imaging

Male BALB/c nude mice (7–10 weeks old; Janvier Labs; Le Genest-Saint-Isle, France) were inoculated subcutaneously with 1 × 10^7^ LNCaP cells suspended in 100 µL PBS:Matrigel (1:1) on the right upper flank. Tumors were allowed to develop until reaching a volume of 300–500 mm^3^. Each ^68^Ga-labeled compound (500 pmol in 0.9% NaCl, 3–6 MBq) was administered via tail vein injection, and animals were anesthetized with 2% isoflurane during image acquisition. Animals were maintained at 37 °C on a temperature-controlled animal bed throughout image acquisition, and respiration and heart rate were continuously monitored (30–60 bpm). Dynamic PET data were acquired over 60 min post injection (p.i.) (30 frames), followed by a non-triggered localizer sequence and a 3-step whole-body T1-weighted 3D MR acquisition using a 3T PET/MR system (Bruker BioSpin GmbH & Co. KG, Ettlingen, Germany). An additional static PET scan (10 min) was performed at 2 h p.i. with subsequent MR imaging using the same protocol. Image reconstruction was performed using ParaVision (version 3.0; Bruker BioSpin GmbH & Co. KG; Ettlingen, Germany) wwith scatter correction (dual energy window), random correction, decay correction, partial volume correction, and point spread function correction enabled for both dynamic and static acquisitions. Dynamic scans were reconstructed using a MAP/MLEM algorithm (0.5 mm isotropic voxels, 12 iterations); static scans were reconstructed using OSEM (0.25 mm isotropic voxels, 1 iteration × 16 subsets) to take advantage of the higher count statistics and enable finer spatial sampling. Attenuation correction was not applied. Quantitative image analysis was performed in PMOD (version 4.1 and 4.3; PMOD Technologies LLC; Zürich, Switzerland).. Volumes of interest (VOIs) for tumor, heart, liver, left and right kidneys, bladder, and muscle were manually delineated on the co-registered T1-weighted MR images by a single observer. Tumor VOIs were additionally refined by isocontour thresholding at 50% of the local maximum voxel value. Dynamic data were analyzed on summed frames for each reported timepoint. Activity concentrations were converted to standardized uptake values normalized to body weight (SUV_bw_) using the injected activity and animal body weight.

#### 4.3.3. Fluorescence Imaging

Following PET/MR imaging, tumor fluorescence was assessed in vivo through intact skin using a custom-built handheld spectroscopic imaging system (Henke-Sass, Wolf GmbH, Tuttlingen, Germany) under isoflurane anesthesia. Subsequently, animals were sacrificed by isoflurane overdose and cervical dislocation.

Ex vivo imaging was performed at a distance of 1 cm against a black background. Images were acquired under white light illumination and excitation light (637 nm) using standardized camera settings. The system operates at a fixed excitation wavelength of 637 nm, matched to the absorption maximum of SulfoCy5; the precise emission filter bandpass is proprietary to the manufacturer and is not disclosed here.

The tumor was imaged sequentially with skin intact, after skin removal, and following excision. Additional organs of interest included heart, lung, spleen, liver, kidneys, intestine, brain, and bone. Leg muscle served as negative control tissue, while kidney was used as a positive control (qualitative). Blood samples were collected to assess circulating fluorescence background. To confirm fluorescent data, all organs were weighed, and radioactivity was measured using a gamma counter (2470 WIZARD_2_ PerkinElmer). Three replicates of 10 µL of the injected compound solution served as an internal standard for dose calculation. Results were expressed as percentage of injected dose per gram tissue (%ID/g). Additionally, all excised organs were transferred to a 24-well plate and imaged at 700 nm and 800 nm using a fluorescence imager (LI-COR Odyssey; LI-COR Biosciences Inc, Lincoln, NE, USA). Three replicates of 10 µL of the injected compound solution served as an internal standard. Imaging was performed *n* = 1.

Quantitative analysis of fluorescence images was performed using a custom ImageJ macro ( version number 1.54p [29], National Institutes of Health, Bethesda, MD, USA). RGB images were processed in batch mode, and the region of image annotations was excluded from the analysis. For signal segmentation, images were split into individual color channels, and intensity thresholding (range: 10–255) was applied to each channel to generate a binary mask distinguishing fluorescent signals from the background. The resulting mask was multiplied with the original image to isolate signal-containing pixels. For each masked image, the following parameters were measured: area, mean fluorescence intensity, minimum and maximum intensity, and integrated density. Cross-compound and cross-tissue comparisons were based on mean fluorescence intensity, which is independent of the segmented signal area and therefore not confounded by differences in tissue or region size.

To establish a background threshold for fluorescence quantification, compounds spectrally incompatible with the endoscopic excitation (*n* = 13) were used as internal background controls. Any signal recorded from these compounds under standardized imaging conditions reflects residual tissue autofluorescence and instrument background rather than dye-specific fluorescence, providing a compound-internal correction for autofluorescence without the need for spectral unmixing. Their mean ± SD was calculated to define a compound-library-specific threshold against which the detectable compounds could be evaluated.

#### 4.3.4. Organ Distribution

Male BALB/c nude mice (7–10 weeks old; Janvier) were inoculated subcutaneously with 1 × 10^7^ LNCaP cells suspended in 100 µL PBS:Matrigel (1:1) on the right upper flank. Tumors were allowed to develop until reaching a volume of 300–500 mm^3^. The respective ^177^Lu-labeled compound (60 pmol in 0.9% NaCl, 0.5–1 MBq) was administered via tail vein injection. Animals were sacrificed by isoflurane overdose at 0.5, 1, and 2 h p.i., and cervical dislocation. Organs of interest (blood, heart, lung, spleen, liver, kidneys, intestine, brain, bone, muscle, and tumor) were excised and weighed. Radioactivity was quantified using a gamma counter (2470 WIZARD_2_ PerkinElmer). Three replicates of 10 µL of the injected compound solution served as the internal standard for dose calculation. Results were expressed as %ID/g. Data represent *n* = 3 animals per time point. Imaging and single-timepoint organ distribution measurements were acquired as single determinations per compound, *n* = 1, and comparisons across the library are therefore presented descriptively rather than by inferential statistical testing.

#### 4.3.5. PSMA Specificity—Negative Control

To confirm PSMA specificity, DP-13 (DP-IRDye800CW) was evaluated in male BALB/c nude inoculated subcutaneously with 5 × 10^6^ PC-3 (PSMA-negative) xenografts. The same experimental protocols as described above were applied, including dynamic PET/MR imaging over 60 min p.i. and static imaging at 2 h p.i. of 500 pmol ^68^Ga-labeled compound, fluorescence imaging using the handheld endoscopic system, organ distribution, and ex vivo fluorescence imaging using the LI-COR Odyssey system.

#### 4.3.6. Artificial Intelligence Assistance

AI tools were used during the preparation of this manuscript in accordance with the TITAN Guideline Checklist 2025 [30]. Claude Opus 4.5 (Anthropic, San Francisco, CA, USA) was employed for two purposes: (1) language editing and improvement of English writing throughout the manuscript, and (2) assistance with Python code development for data visualization and plotting. All AI-generated outputs were critically reviewed, verified against primary experimental data, and edited by the authors. Only de-identified experimental data were provided as input; no patient data were involved. The authors take full responsibility for the accuracy and integrity of all content.

## 5. Conclusions

Fluorescence evaluation using a clinical-grade endoscopic system identified DP-12 (DP-SulfoCy5), DP-15 (DP-Alexa Fluor 647), and DP-18 (DP-Tide Fluor 5WS) as lead candidates for clinical translation within the same-day surgical workflow targeted here, combining high tumor-to-muscle contrast at the surgical timepoint, with ratios of 9.5, 38.6, and 21.7, respectively, alongside rapid kidney washout and spectral compatibility with the current endoscopic setup. Beyond these lead candidates, compounds with distinctive spectral properties, such as the large Stokes shift dyes DP-19 (DP-Atto490LS) or DP-24 (DP-mFluor Blue 660 SE), warrant further investigation, as they could offer improved signal separation once suitable imaging hardware becomes available. These findings derive from a single subcutaneous tumor model evaluated within a same-day imaging window and without direct intraoperative surgical validation. Building on this descriptive screen, the most promising candidates will therefore be taken forward into focused studies with fewer compounds and higher replication, in which statistically powered comparisons can be made and the leads can be validated in more complex models and in an intraoperative surgical setting, before clinical translation.

## Figures and Tables

**Table 1:** Selected fluorescent dyes used in this study. Nomenclature follows the scheme DP-#, denoting conjugates of the precursor DP with the respective fluorescent dye. Absorption (Abs.) and emission (Em.) maxima are given in nm. Molecular weights are reported for both the free dye (MW_Dye_; free acid form without NHS-ester) and the final peptide–dye conjugate (MW_Conjugate_). Net charge (z) refers to the dye moiety at physiological pH.

Name	Conjugate	Abs. Max. [nm]	Em. Max. [nm]	MW_Dye_ [g/mol]	MW_Conjugate_ [g/mol]	z
DP-1	DP-Atto590	594	624	573.29	2879.88	0
DP-2	DP-Alexa594	590	618	703.84	3010.43	−2
DP-4	DP-STAR635P	638	651	915.85	3222.44	−1
DP-5	DP-STAR RED	638	655	886.02	3192.61	−1
DP-8	DP-CF680R	680	701	895.15	3201.74	−2
DP-9	DP-580CP-Methoxy	582	607	441.59	2748.18	0
DP-10	DP-610CP	609	634	439.62	2746.21	0
DP-11	DP-Sir-Methyl	648	662	426.71	2733.3	+1
DP-12	DP-SulfoCy5	646	662	624.85	2931.44	−1
DP-13	DP-IRDye800CW	774	789	983.23	3289.82	−3
DP-15	DP-Alexa647	650	671	825.03	3131.62	−3
DP-16	DP-SulfoCy7	750	773	690.95	2997.54	−1
DP-17	DP-SulfoCy7.5	778	797	949.17	3255.76	−3
DP-18	DP-Tide Fluor5WS	649	663	732	3038.59	−2
DP-19	DP-Atto490LS	495	663	679	2985.59	−1
DP-20	DP-DY-521XL	533	674	590.74	2897.33	−1
DP-22	DP-DY-647	653	672	624.85	2931.44	−1
DP-23	DP-DY-540XL	537	624	743.92	3050.51	−2
DP-24	DP-mFluor^TM^ Blue 660 SE	481	663	532.71	2839.3	−1

**Table 2:** In vitro characterization of dual-labeled PSMA-targeting compounds. Values are given as mean ± standard deviation (SD) of three independent experiments, except serum stability, which represents single determinations (*n* = 1). LogD_pH 7.4_ (*n* = 3); serum stability in human and murine serum after 24 h (%, *n* = 1); PSMA-binding affinity (K_i_, nM, *n* = 3); cell surface binding and internalization in LNCaP cells (%AA/10^5^ cells, *n* = 3).

	logD_pH7.4_	Serum Stability After 24 h [%]		K_i_ [nM]	Internalization Assay [%AA/10^5^ Cells]	
		Human	Murine		Surface Bound	Internalized
DP-1	−1.87 ± 0.13	97.92	97.24	74.97 ± 18.65	0.44 ± 0.01	0.51 ± 0.02
DP-2	−2.65 ± 0.03	100	96.51	23.31 ± 2.76	0.36 ± 0.08	0.51 ± 0.07
DP-4	−2.25 ± 0.10	73.4	9.92	27.43 ± 1.40	0.35 ± 0.21	0.34 ± 0.23
DP-5	−2.34 ± 0.16	98.22	76.71	83.02 ± 40.51	0.17 ± 0.01	0.22 ± 0.02
DP-8	−2.48 ± 0.06	100	97.31	51.77 ± 10.67	0.33 ± 0.13	0.29 ± 0.04
DP-9	−1.99 ± 0.37	100	93.07	19.35 ± 0.31	0.20 ± 0.04	0.25 ± 0.05
DP-10	−2.27 ± 0.06	94.5	92.87	29.64 ± 3.26	0.53 ± 0.28	0.32 ± 0.08
DP-11	−2.21 ± 0.17	96.69	13.26	86.89 ± 20.20	0.30 ± 0.06	0.31 ± 0.10
DP-12	−2.62 ± 0.02	98.87	98.21	38.76 ± 10.33	0.39 ± 0.04	0.18 ± 0.07
DP-13	−2.15 ± 0.10	47.53	53.79	19.20 ± 4.87	0.08 ± 0.02	0.40 ± 0.06
DP-15	−2.46 ± 0.04	100	100	34.96 ± 12.41	0.07 ± 0.01	0.32 ± 0.02
DP-16	−2.67 ± 0.09	98.57	96.08	32.23 ± 5.80	0.57 ± 0.03	0.16 ± 0.11
DP-18	−2.74 ± 0.05	100	100	35.30 ± 2.64	0.26 ± 0.05	0.07 ± 0.16
DP-19	−3.02 ± 0.37	100	96.2	32.19 ± 4.15	0.12 ± 0.01	0.26 ± 0.01
DP-20	−3.72 ± 0.23	94.88	89.14	22.09 ± 5.33	0.15 ± 0.05	0.32 ± 0.08
DP-22	−2.97 ± 0.99	100	97.74	38.07 ± 5.62	0.19 ± 0.05	0.31 ± 0.06
DP-23	−2.42 ± 1.24	100	97.61	18.03 ± 2.93	0.09 ± 0.02	0.47 ± 0.07
DP-24	−1.79 ± 0.45	97.77	100	23.28 ± 2.52	0.18 ± 0.04	0.79 ± 0.15

**Table 3:** Organ distribution (%ID/g) at 2 h p.i. following PET/MR and fluorescence imaging (500 pmol compound). Kidney values represent the mean of both kidneys, except those indicated with *, which represent a single kidney. All values represent single measurements (*n* = 1).

Compound	Blood	Spleen	Liver	Kidney	Muscle	Tumor
DP-1	0.99	0.48	0.45	18.74	0.11	8.33
DP-2	0.21	0.40	0.13	31.21	0.04	6.59
DP-4	0.27	0.17	0.18	12.68	0.04	3.26
DP-5	0.69	0.31	0.30	18.04	0.13	7.31
DP-8	0.09	0.09	0.05	4.71	0.03	1.92
DP-9	0.24	0.23	0.14	26.27	0.68	9.14
DP-10	0.16	0.16	0.08	4.76	0.03	4.13
DP-11	0.20	0.34	0.59	3.33 *	0.26	1.24
DP-12	0.46	0.37	0.22	8.80	0.12	11.11
DP-13	0.49	0.62	0.42	46.56	0.10	6.53
DP-15	0.13	0.19	0.08	7.68	0.03	7.67
DP-16	0.77	0.61	0.40	24.43	0.09	7.13
DP-18	0.25	0.57	0.20	15.85	0.06	8.77
DP-19	0.40	0.42	0.32	28.59	0.08	8.17
DP-20	0.37	0.42	0.15	13.51	0.08	1.68
DP-22	0.37	0.35	0.16	19.48	0.05	6.26
DP-23	0.36	0.48	0.23	81.94	0.05	5.69
DP-24	4.81	3.52	1.43	61.97	0.67	22.94

## Data Availability

Complete organ distribution data, serum stability time courses, and all raw numerical data supporting the findings of this study are available from the corresponding author upon reasonable request.

## Supplementary Materials

The following supporting information can be downloaded at: https://www.mdpi.com/article/10.3390/ph19081187/s1, Figure S1: Exemplary analytical RP-HPLC chromatogram of DP-12 (DP-SulfoCy5); Figure S2: Exemplary MALDI-TOF mass spectrum of DP-12 (DP-SulfoCy5); Figure S3: IC50 curves from competitive binding assays; Figure S4: Internalization assay data with blocking and temperature controls; Figure S5: PET/MR maximum intensity projection images at 1 h p.i. for all dual-labeled compounds; Figure S6: Dynamic time–activity curves for tumor, muscle, and kidneys; Figure S7: PET-derived tumor-to-muscle ratio time courses; Figure S8: Ex vivo LI-COR fluorescence images at 700 and 800 nm; Figure S9: PSMA specificity data for DP-13 in PC-3 (PSMA-negative) xenograft model.

## Abbreviations

The following abbreviations are used in this manuscript:

%AA	Percentage of applied activity
%ID/g	Percentage of injected dose per gram
2-PMPA	2-(phosphonomethyl)pentanedioic acid
DMSO	Dimethyl sulfoxide
DOTA	1,4,7,10-tetraazacyclododecane-1,4,7,10-tetraacetic acid
EuK	Glutamate-urea-lysine
FGS	Fluorescence-guided surgery
HEPES	4-(2-hydroxyethyl)-1-piperazineethanesulfonic acid
IC_50_	Half maximal inhibitory concentration
ICG	Indocyanine green
K_d_	Dissociation constant
K_i_	Inhibition constant
logD	Distribution coefficient (lipophilicity)
MALDI-TOF-MS	Matrix-assisted laser desorption/ionization time-of-flight mass spectrometry
MIP	Maximum intensity projection
MLEM	Maximum-Likelihood Expectation-Maximization
MR/MRI	Magnetic resonance imaging
MW	Molecular weight
NHS	N-hydroxysuccinimide
NIR	Near-infrared
OSEM	Ordered subset expectation maximization
p.i.	Post injection
PBS	Phosphate-buffered saline
PC	Prostate cancer
PET	Positron emission tomography
PSMA	Prostate-specific membrane antigen
RGS	Radio-guided surgery
RLT	Radioligand therapy
RP	Radical prostatectomy
RP-HPLC	Reversed-phase high-performance liquid chromatography
RT	Room temperature
SCX	Strong Cation Exchange
SD	Standard deviation
STED	Stimulated emission depletion
SUV_bw_	Standardized uptake values normalized to body weight
TAC	Time–activity curves
TFA	Trifluoroacetic acid

## References

[1] Schafer E.J., Laversanne M., Sung H., Soerjomataram I., Briganti A., Dahut W., Bray F., Jemal A. (2025). Recent Patterns and Trends in Global Prostate Cancer Incidence and Mortality: An Update. Eur. Urol..

[2] Bray F., Laversanne M., Sung H., Ferlay J., Siegel R.L., Soerjomataram I., Jemal A. (2024). Global Cancer Statistics 2022: GLOBOCAN Estimates of Incidence and Mortality Worldwide for 36 Cancers in 185 Countries. CA Cancer J. Clin..

[3] Cornford P., Van Den Bergh R.C.N., Briers E., Van Den Broeck T., Brunckhorst O., Darraugh J., Eberli D., De Meerleer G., De Santis M., Farolfi A. (2024). EAU-EANM-ESTRO-ESUR-ISUP-SIOG Guidelines on Prostate Cancer—2024 Update. Part I: Screening, Diagnosis, and Local Treatment with Curative Intent. Eur. Urol..

[4] Beckmann K.R., O’Callaghan M.E., Vincent A.D., Moretti K.L., Brook N.R. (2023). Clinical Outcomes for Men with Positive Surgical Margins after Radical Prostatectomy—Results from the South Australian Prostate Cancer Clinical Outcomes Collaborative Community-Based Registry. Asian J. Urol..

[5] Stephenson A.J., Eggener S.E., Hernandez A.V., Klein E.A., Kattan M.W., Wood D.P., Rabah D.M., Eastham J.A., Scardino P.T. (2014). Do Margins Matter? The Influence of Positive Surgical Margins on Prostate Cancer–Specific Mortality. Eur. Urol..

[6] Pellegrino F., Falagario U.G., Knipper S., Martini A., Akre O., Egevad L., Aly M., Moschovas M.C., Bravi C.A., Tran J. (2024). Assessing the Impact of Positive Surgical Margins on Mortality in Patients Who Underwent Robotic Radical Prostatectomy: 20 Years’ Report from the EAU Robotic Urology Section Scientific Working Group. Eur. Urol. Oncol..

[7] Derks Y.H.W., Löwik D.W.P.M., Sedelaar J.P.M., Gotthardt M., Boerman O.C., Rijpkema M., Lütje S., Heskamp S. (2019). PSMA-Targeting Agents for Radio- and Fluorescence-Guided Prostate Cancer Surgery. Theranostics.

[8] Maurer T., Eiber M., Schwaiger M., Gschwend J.E. (2016). Current Use of PSMA–PET in Prostate Cancer Management. Nat. Rev. Urol..

[9] Eder M., Neels O., Müller M., Bauder-Wüst U., Remde Y., Schäfer M., Hennrich U., Eisenhut M., Afshar-Oromieh A., Haberkorn U. (2014). Novel Preclinical and Radiopharmaceutical Aspects of [68Ga]Ga-PSMA-HBED-CC: A New PET Tracer for Imaging of Prostate Cancer. Pharmaceuticals.

[10] Sartor O., De Bono J., Chi K.N., Fizazi K., Herrmann K., Rahbar K., Tagawa S.T., Nordquist L.T., Vaishampayan N., El-Haddad G. (2021). Lutetium-177–PSMA-617 for Metastatic Castration-Resistant Prostate Cancer. N. Engl. J. Med..

[11] Eder A.-C., Matthias J., Schäfer M., Schmidt J., Steinacker N., Bauder-Wüst U., Domogalla L.-C., Roscher M., Haberkorn U., Eder M. (2022). A New Class of PSMA-617-Based Hybrid Molecules for Preoperative Imaging and Intraoperative Fluorescence Navigation of Prostate Cancer. Pharmaceuticals.

[12] Mix M., Schultze-Seemann W., Von Büren M., Sigle A., Omrane M.A., Grabbert M.T., Werner M., Gratzke C., Meyer P.T., Jilg C.A. (2021). 99mTc-Labelled PSMA Ligand for Radio-Guided Surgery in Nodal Metastatic Prostate Cancer: Proof of Principle. EJNMMI Res..

[13] Minges P., Eder M., Eder A.-C. (2025). Dual-Labeled Small Peptides in Cancer Imaging and Fluorescence-Guided Surgery: Progress and Future Perspectives. Pharmaceuticals.

[14] Nagaya T., Nakamura Y.A., Choyke P.L., Kobayashi H. (2017). Fluorescence-Guided Surgery. Front. Oncol..

[15] Eder A.-C., Schäfer M., Schmidt J., Bauder-Wüst U., Roscher M., Leotta K., Haberkorn U., Kopka K., Eder M. (2021). Rational Linker Design to Accelerate Excretion and Reduce Background Uptake of Peptidomimetic PSMA-Targeting Hybrid Molecules. J. Nucl. Med..

[16] Herth M.M., Ametamey S., Antuganov D., Bauman A., Berndt M., Brooks A.F., Bormans G., Choe Y.S., Gillings N., Häfeli U.O. (2021). On the Consensus Nomenclature Rules for Radiopharmaceutical Chemistry—Reconsideration of Radiochemical Conversion. Nucl. Med. Biol..

[17] Li P., Xu Y., Yu S., Miao Z., Xu H., Lu B., Zhou S., Gan W., Couñago F., Kairemo K. (2025). First-in-Human Study of DGPR1008 for Intraoperative Fluorescence Imaging of Prostate-Specific Membrane Antigen-Positive Prostate Cancer in Patients Undergoing Radical Prostatectomy. Transl. Androl. Urol..

[18] Eder A.-C., Omrane M.A., Stadlbauer S., Roscher M., Khoder W.Y., Gratzke C., Kopka K., Eder M., Meyer P.T., Jilg C.A. (2021). The PSMA-11-Derived Hybrid Molecule PSMA-914 Specifically Identifies Prostate Cancer by Preoperative PET/CT and Intraoperative Fluorescence Imaging. Eur. J. Nucl. Med. Mol. Imaging.

[19] Schottelius M., Viertl D., Buckle T., Koch H., Martin S., Litvinenko A., Patt M., Van Willigen D.M., Van Leeuwen F.W.B., Wester H.-J. (2025). [99mTc]Tc-PSMA-HSG for PSMA-Targeted Hybrid Surgical Guidance: A New Addition to the PSMA-I&S/I&T Family. Eur. J. Nucl. Med. Mol. Imaging.

[20] Benešová M., Schäfer M., Bauder-Wüst U., Afshar-Oromieh A., Kratochwil C., Mier W., Haberkorn U., Kopka K., Eder M. (2015). Preclinical Evaluation of a Tailor-Made DOTA-Conjugated PSMA Inhibitor with Optimized Linker Moiety for Imaging and Endoradiotherapy of Prostate Cancer. J. Nucl. Med..

[21] Afshar-Oromieh A., Hetzheim H., Kratochwil C., Benesova M., Eder M., Neels O.C., Eisenhut M., Kübler W., Holland-Letz T., Giesel F.L. (2015). The Theranostic PSMA Ligand PSMA-617 in the Diagnosis of Prostate Cancer by PET/CT: Biodistribution in Humans, Radiation Dosimetry, and First Evaluation of Tumor Lesions. J. Nucl. Med..

[22] Mu H., Shao S., Wu B., Miki K., Kobayashi M., Harada H., Ohe K. (2024). Fast Tumor Imaging Using pH-Responsive Aggregation of Cyanine Dyes with Rapid Clearance. Sens. Actuators B Chem..

[23] Zhu N., Xu J., Su Q., Han T., Zhou D., Zhang Y., Zhu S. (2024). Site-Specific Albumin Tagging with NIR-II Fluorogenic Dye for High-Performance and Super-Stable Bioimaging. Theranostics.

[24] Zhegalova N.G., He S., Zhou H., Kim D.M., Berezin M.Y. (2014). Minimization of Self-quenching Fluorescence on Dyes Conjugated to Biomolecules with Multiple Labeling Sites via Asymmetrically Charged NIR Fluorophores. Contrast Media Mol..

[25] Lee Y.-J., Van Den Berg N.S., Orosco R.K., Rosenthal E.L., Sorger J.M. (2021). A Narrative Review of Fluorescence Imaging in Robotic-Assisted Surgery. Laparosc. Surg..

[26] Vilhelmsson Timmermand O., Safi M., Holmqvist B., Strand J. (2023). Evaluation of Enhanced Permeability Effect and Different Linear Energy Transfer of Radionuclides in a Prostate Cancer Xenograft Model. Am. J. Nucl. Med. Mol. Imaging.

[27] Vahrmeijer A.L., Hutteman M., Van Der Vorst J.R., Van De Velde C.J.H., Frangioni J.V. (2013). Image-Guided Cancer Surgery Using near-Infrared Fluorescence. Nat. Rev. Clin. Oncol..

[28] Schäfer M., Bauder-Wüst U., Leotta K., Zoller F., Mier W., Haberkorn U., Eisenhut M., Eder M. (2012). A Dimerized Urea-Based Inhibitor of the Prostate-Specific Membrane Antigen for 68Ga-PET Imaging of Prostate Cancer. EJNMMI Res..

[29] Schneider C.A., Rasband W.S., Eliceiri K.W. (2012). NIH Image to ImageJ: 25 Years of Image Analysis. Nat. Methods.

[30] Agha R.A., Mathew G., Rashid R., Kerwan A., Al-Jabir A., Sohrabi C., Franchi T., Nicola M., Agha M. (2025). Transparency in the Reporting of Artificial Intelligence—The TITAN Guideline. Prem. J. Sci..

