# Supplementary material for: Sarcosine-Based Pharmacokinetic Optimization and Fluorescent Dye Library Evaluation of Dual-Labeled PSMA Inhibitors for Fluorescence-Guided Surgery
Source: Pharmaceuticals (Basel). 2026 Jul 29;19(8):1187. doi: 10.3390/ph19081187 (PMC13516764; doi:10.3390/ph19081187)

Supplementary Figure S 1: Exemplary analytical RP-HPLC chromatogram used for confirming chemical purity of all dual-labeled conjugates. Analysis was performed using a Chromolith® Performance RP-18e column (100 × 4.6 mm) with a gradient from 90% water/0.1% TFA to 90% acetonitrile/0.1% TFA over 15 min. All compounds exhibited chemical purity >95% prior to subsequent experiments. UV detection was performed at 214 nm and 280 nm. Shown is for example DP-12 (DP-SulfoCy5)..3
Supplementary Figure S 2: Exemplary MALDI-TOF mass spectrum confirming molecular identity of DP-12 (DP-SulfoCy5) with calculated mass 2931.35 m/z. Spectrum was acquired in positive ion mode. All observed masses matched calculated values within instrument accuracy. detected as either $[M+H]^+$ or $[M+Na]^+$ adducts. 4
Supplementary Figure S 3: IC_{50} curves from competitive binding assays. Binding affinities were determined in LNCaP cells using [^{68}Ga]Ga-PSMA-10 as reference radioligand (k_d = 3.8 ± 1.8 nM). Serial dilutions of cold test compounds (0–5000 nM) were co-incubated with 150 nM [^{68}Ga]Ga-PSMA-10 for 1 h at 37 °C. IC_{50} values were calculated by nonlinear regression analysis, and inhibition constants (k_i) were derived using the Cheng-Prusoff equation. Data represent mean ± SD (n = 3 biological replicates, each measured in 4 technical replicates). 5
Supplementary Figure S 4: Complete internalization assay data including blocking controls. ^{177}Lu-labeled compounds (30 nM) were incubated with LNCaP cells for 45 min at 37 °C or 4 °C. Surface-bound activity was removed by acid wash (glycine-HCl buffer). and internalized activity was collected by cell lysis (0.3 M NaOH). Specificity was confirmed by co-incubation with 500 µM 2-PMPA. ATP-dependent internalization was verified by parallel incubation at 4 °C. Results are expressed as specific activity (%) per 10^5 cells. Data represent mean ± SD (n = 3 biological replicates. each measured in 3 technical replicates). 6
Supplementary Figure S 5: PET/MR maximum intensity projection (MIP) images for all dual-labeled compounds at 1 h p.i. following injection of 500 pmol ^{68}Ga-labeled compound in LNCaP tumor-bearing mice. Color scale represents SUV_{bw}. Images are displayed with MIP 1.8...7
Supplementary Figure S 6: Dynamic time-activity curves (TACs) for all 19 dual-labeled compounds derived from PET imaging following administration of 500 pmol of the respective ^{68}Ga-labeled compound in LNCaP tumor-bearing mice. Each panel shows a single compound on shared time (0–60 min + 120 min p.i.) and SUV_{bw} axes. A) Tumor (blue) and muscle (green), B) Kidney uptake (red). 8
Supplementary Figure S 7: Tumor-to-muscle (T/M) ratios were calculated from PET-derived standardized uptake values normalized to body weight (SUV_{bw}). Data represent dynamic scans over the first 60 min p.i. (30 frames) and static scans at 120 min p.i., following administration of 500 pmol of corresponding ^{68}Ga-labeled compound. 8
Supplementary Figure S 8: *Ex vivo* fluorescence imaging using LI-COR Odyssey system. Following PET/MR imaging at 2 h p.i., organs were excised and imaged at 700 nm and 800 nm. Large Stokes shift dyes (DP-19: DP-Atto490LS, DP-20: DP-DY-521XL, DP-23: DP-DY-540XL, DP-24: DP-mFluor Blue 660 SE) could not be assessed with this system due to incompatible excitation wavelengths. Signal intensity is displayed in

arbitrary units (a.u.). All images within each channel are displayed with identical settings for direct comparison. 9

Supplementary Figure S 9: PSMA specificity confirmed in PC-3 (PSMA-negative) xenograft model. Following administration of 500 pmol ^{68}Ga-labeled compound of DP-13 (DP-IRDye800CW) in PC-3 tumor-bearing BALB/c nude mice. (A) PET/MR maximum intensity projection (MIP) images at 1 h & 2 h p.i., (B) fluorescence imaging (excitation 637 nm) of kidney (positive control), muscle (negative control), and PC-3 tumor, (C) radioactive organ distribution (%ID/g) at 2 h p.i., and (D) *ex vivo* fluorescence imaging using LI-COR Odyssey system, measuring at 800 nm. 10

Supplementary Figure S 1: Exemplary analytical RP-HPLC chromatogram used for confirming chemical purity of all dual-labeled conjugates. Analysis was performed using a Chromolith® Performance RP-18e column (100 × 4.6 mm) with a gradient from 90% water/0.1% TFA to 90% acetonitrile/0.1% TFA over 15 min. All compounds exhibited chemical purity >95% prior to subsequent experiments. UV detection was performed at 214 nm and 280 nm. Shown is for example DP-12 (DP-SulfoCy5).

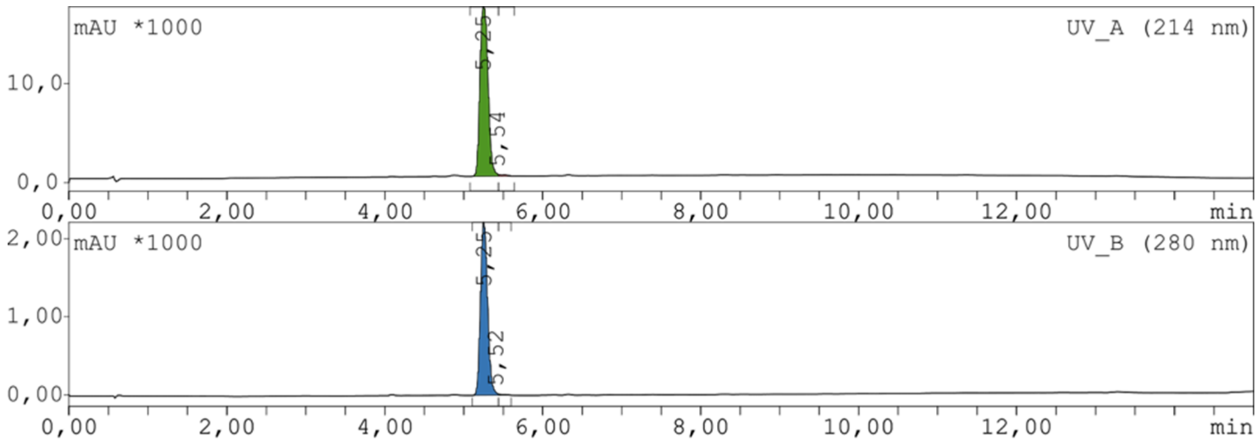

UV_A (214 nm)

Substance	R/T min	Type	Area mAU*min	%Area %	Sig-Noise	Start min	Stop min
Reg #1	5,25	BD	120109,8	99,21	0,0	5,08	5,44
Reg #2	5,54	DB	951,8	0,79	0,0	5,44	5,64
Sum in ROI			121061,6				

UV_B (280 nm)

Substance	R/T min	Type	Area mAU*min	%Area %	Sig-Noise	Start min	Stop min
Reg #1	5,25	BD	13509,46	99,49	0,0	5,11	5,44
Reg #2	5,52	DB	68,62	0,51	0,0	5,44	5,60
Sum in ROI			13578,08				

Supplementary Figure S 2: Exemplary MALDI-TOF mass spectrum confirming molecular identity of DP-12 (DP-SulfoCy5) with calculated mass 2931.35 m/z. Spectrum was acquired in positive ion mode. All observed masses matched calculated values within instrument accuracy. detected as either $[M+H]^+$ or $[M+Na]^+$ adducts.

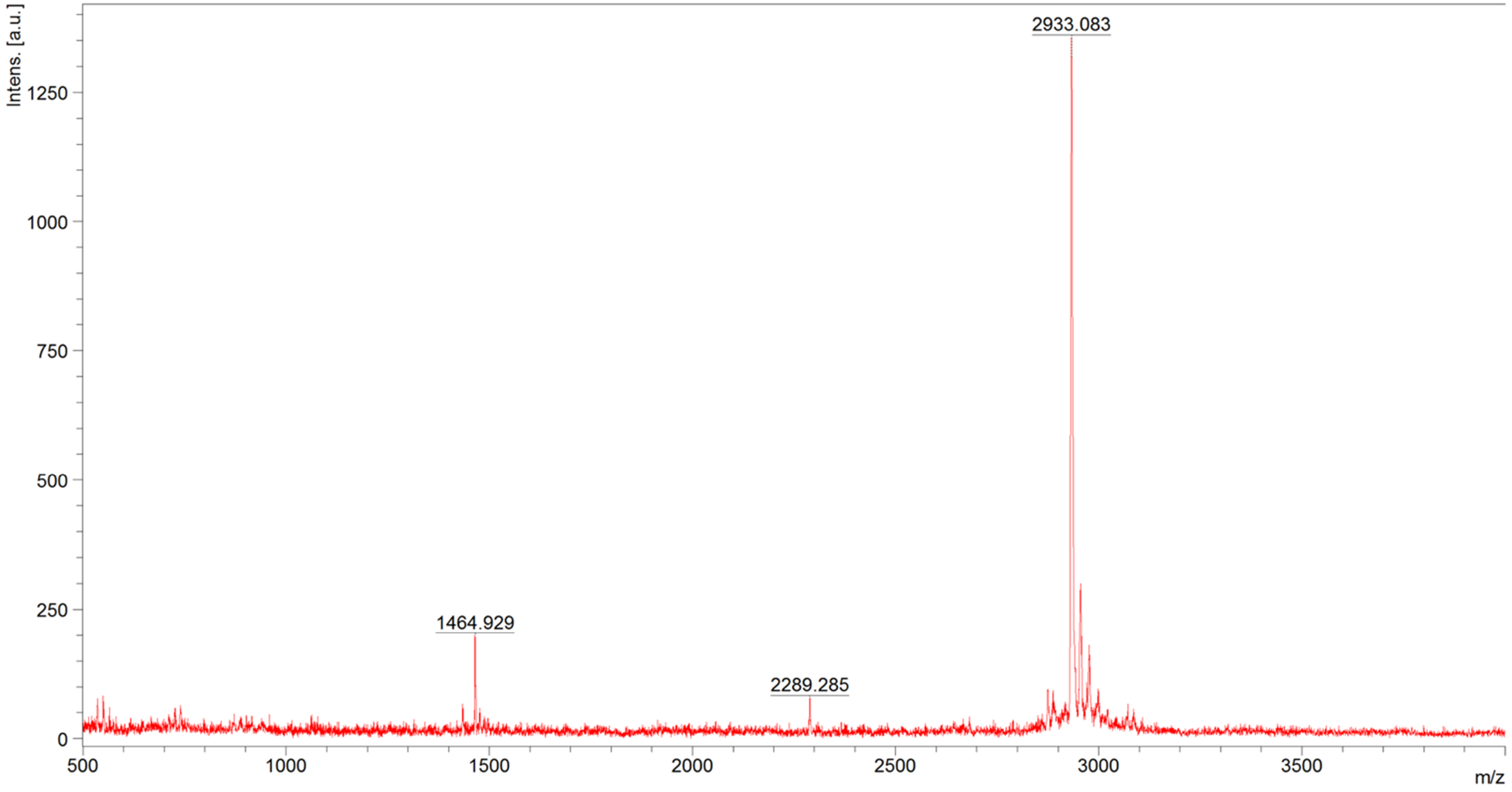

Supplementary Figure S 3: IC_{50} curves from competitive binding assays. Binding affinities were determined in LNCaP cells using [^{68}Ga]Ga-PSMA-10 as reference radioligand (k_d = 3.8 ± 1.8 nM). Serial dilutions of cold test compounds (0–5000 nM) were co-incubated with 150 nM [^{68}Ga]Ga-PSMA-10 for 1 h at 37 °C. IC_{50} values were calculated by nonlinear regression analysis, and inhibition constants (k_i) were derived using the Cheng-Prusoff equation. Data represent mean ± SD (n = 3 biological replicates, each measured in 4 technical replicates).

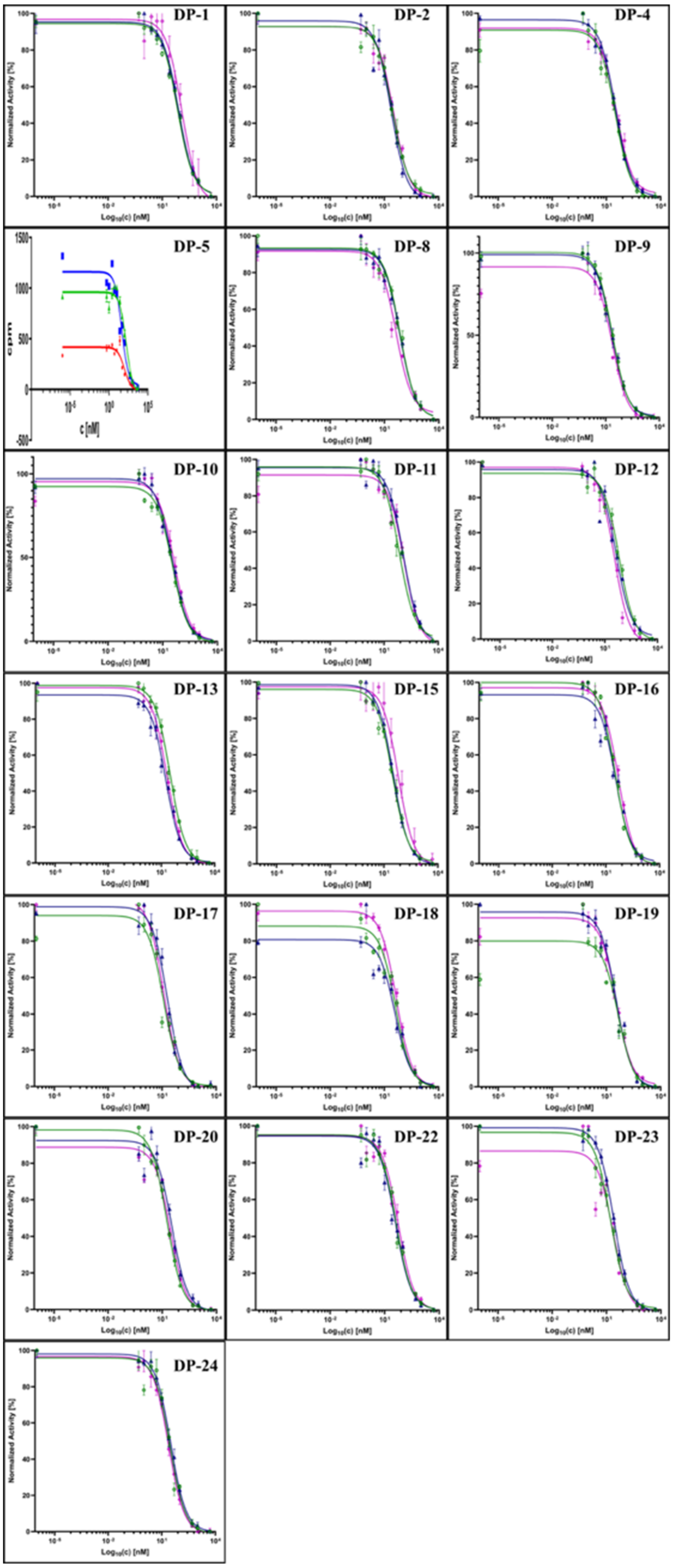

Supplementary Figure S 4: Complete internalization assay data including blocking controls. ^{177}Lu-labeled compounds (30 nM) were incubated with LNCaP cells for 45 min at 37 °C or 4 °C. Surface-bound activity was removed by acid wash (glycine-HCl buffer). and internalized activity was collected by cell lysis (0.3 M NaOH). Specificity was confirmed by co-incubation with 500 µM 2-PMPA. ATP-dependent internalization was verified by parallel incubation at 4 °C. Results are expressed as specific activity (%) per 10^5 cells. Data represent mean ± SD (n = 3 biological replicates. each measured in 3 technical replicates).

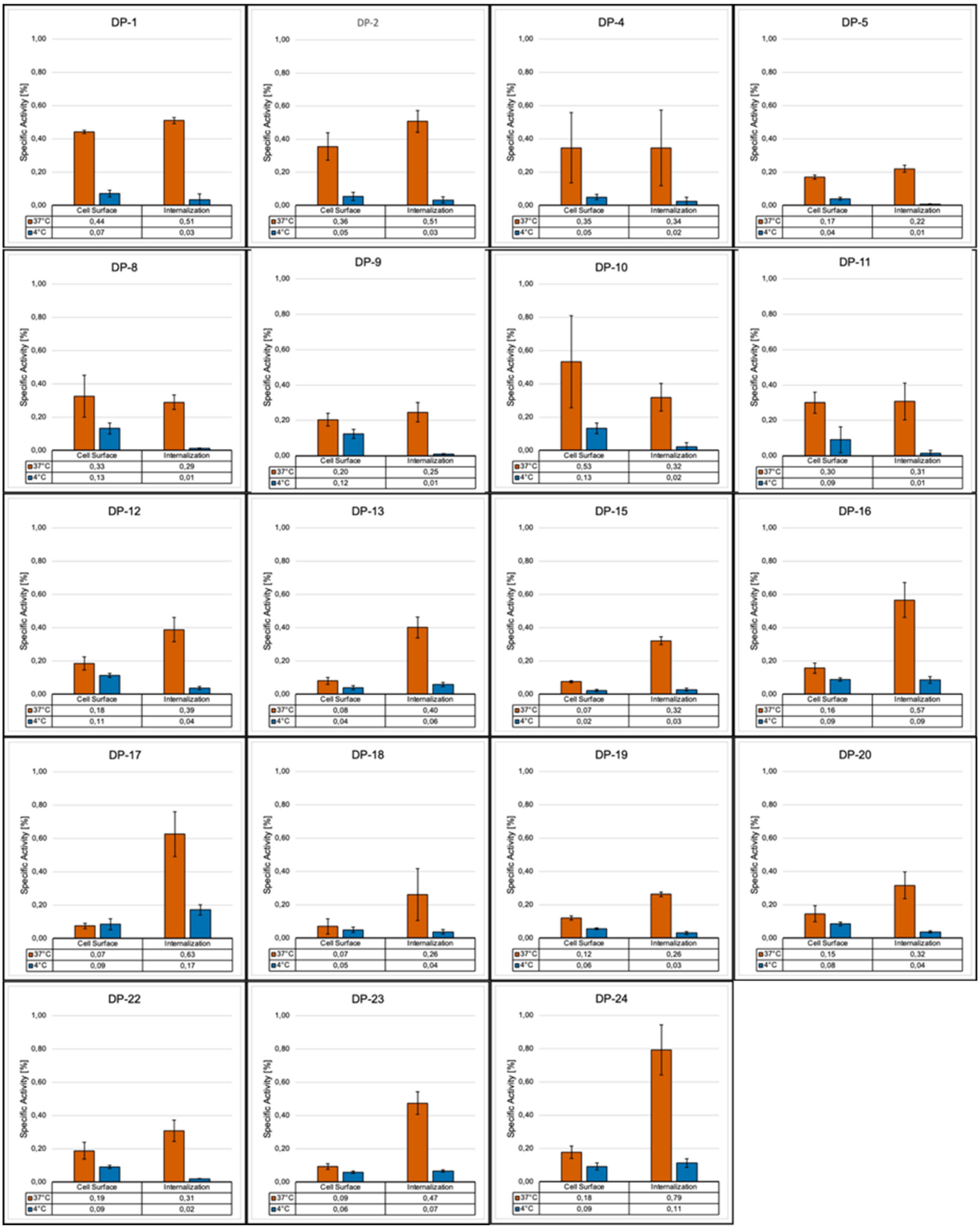

Supplementary Figure S 5: PET/MR maximum intensity projection (MIP) images for all dual-labeled compounds at 1 h p.i. following injection of 500 pmol ^{68}Ga-labeled compound in LNCaP tumor-bearing mice. Color scale represents SUV_{bw}. Images are displayed with MIP 1.8.

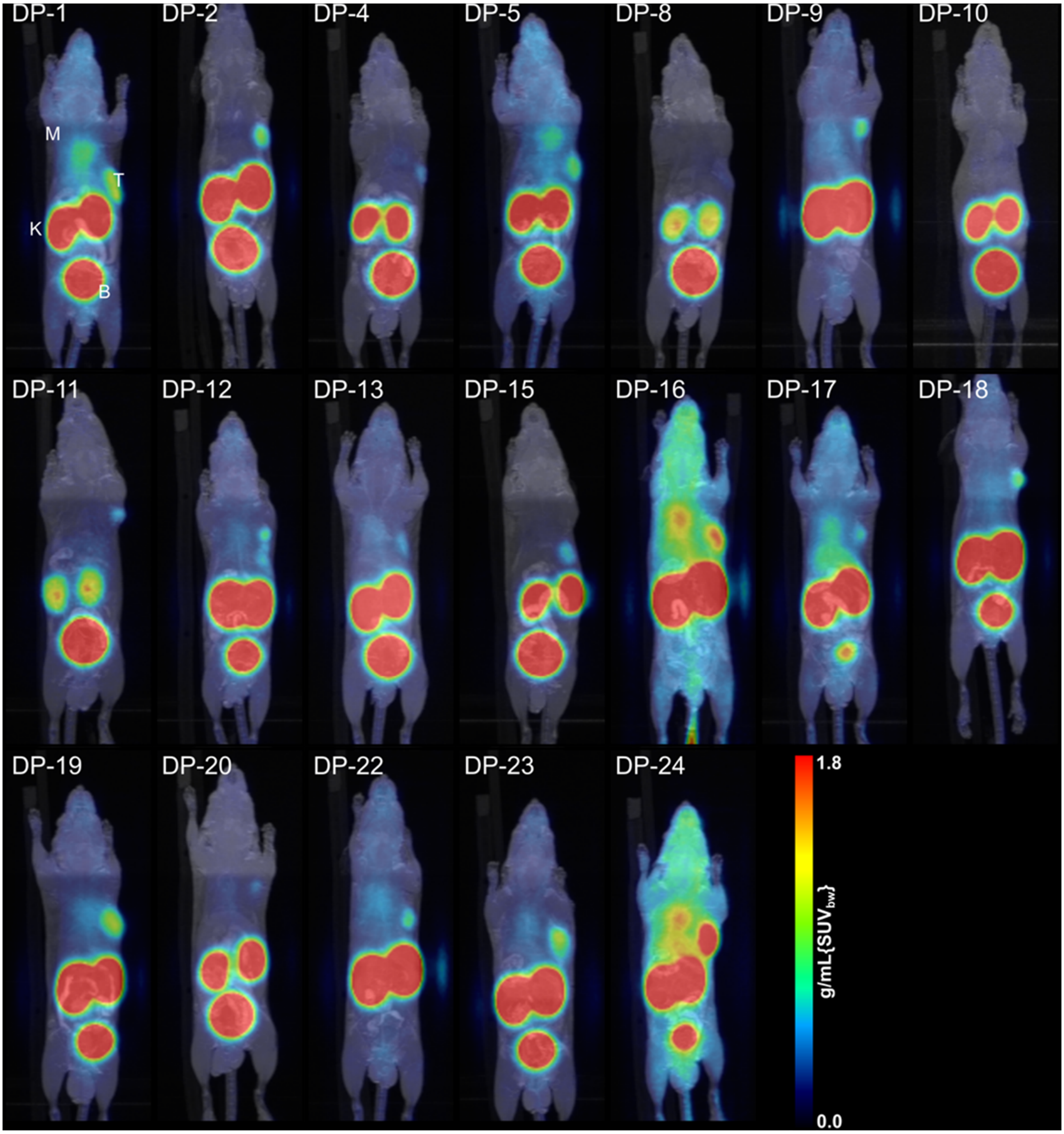

Supplementary Figure S 6: Dynamic time-activity curves (TACs) for all 19 dual-labeled compounds derived from PET imaging following administration of 500 pmol of the respective ^{68}Ga-labeled compound in LNCaP tumor-bearing mice. Each panel shows a single compound on shared time (0–60 min + 120 min p.i.) and SUV_{bw} axes. A) Tumor (blue) and muscle (green), B) Kidney uptake (red).

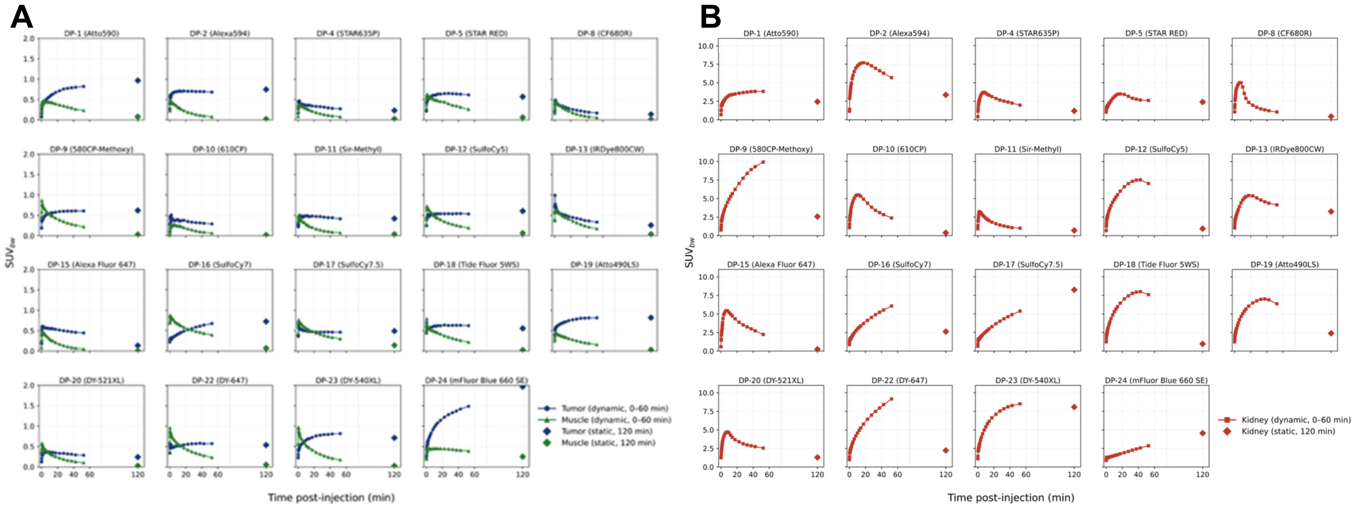

Supplementary Figure S 7: Tumor-to-muscle (T/M) ratios were calculated from PET-derived standardized uptake values normalized to body weight (SUV_{bw}). Data represent dynamic scans over the first 60 min p.i. (30 frames) and static scans at 120 min p.i., following administration of 500 pmol of corresponding ^{68}Ga-labeled compound.

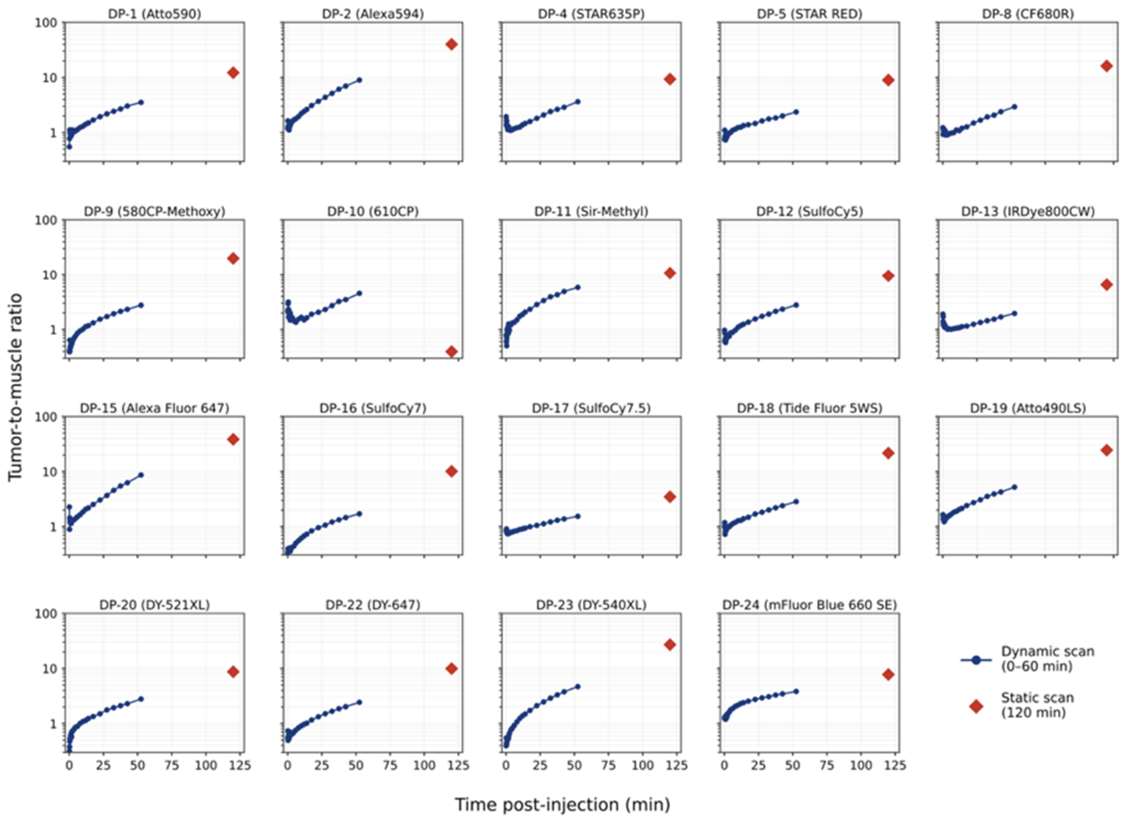

Supplementary Figure S 8: *Ex vivo* fluorescence imaging using LI-COR Odyssey system. Following PET/MR imaging at 2 h p.i., organs were excised and imaged at 700 nm and 800 nm. Large Stokes shift dyes (DP-19: DP-Atto490LS, DP-20: DP-DY-521XL, DP-23: DP-DY-540XL, DP-24: DP-mFluor Blue 660 SE) could not be assessed with this system due to incompatible excitation wavelengths. Signal intensity is displayed in arbitrary units (a.u.). All images within each channel are displayed with identical settings for direct comparison.

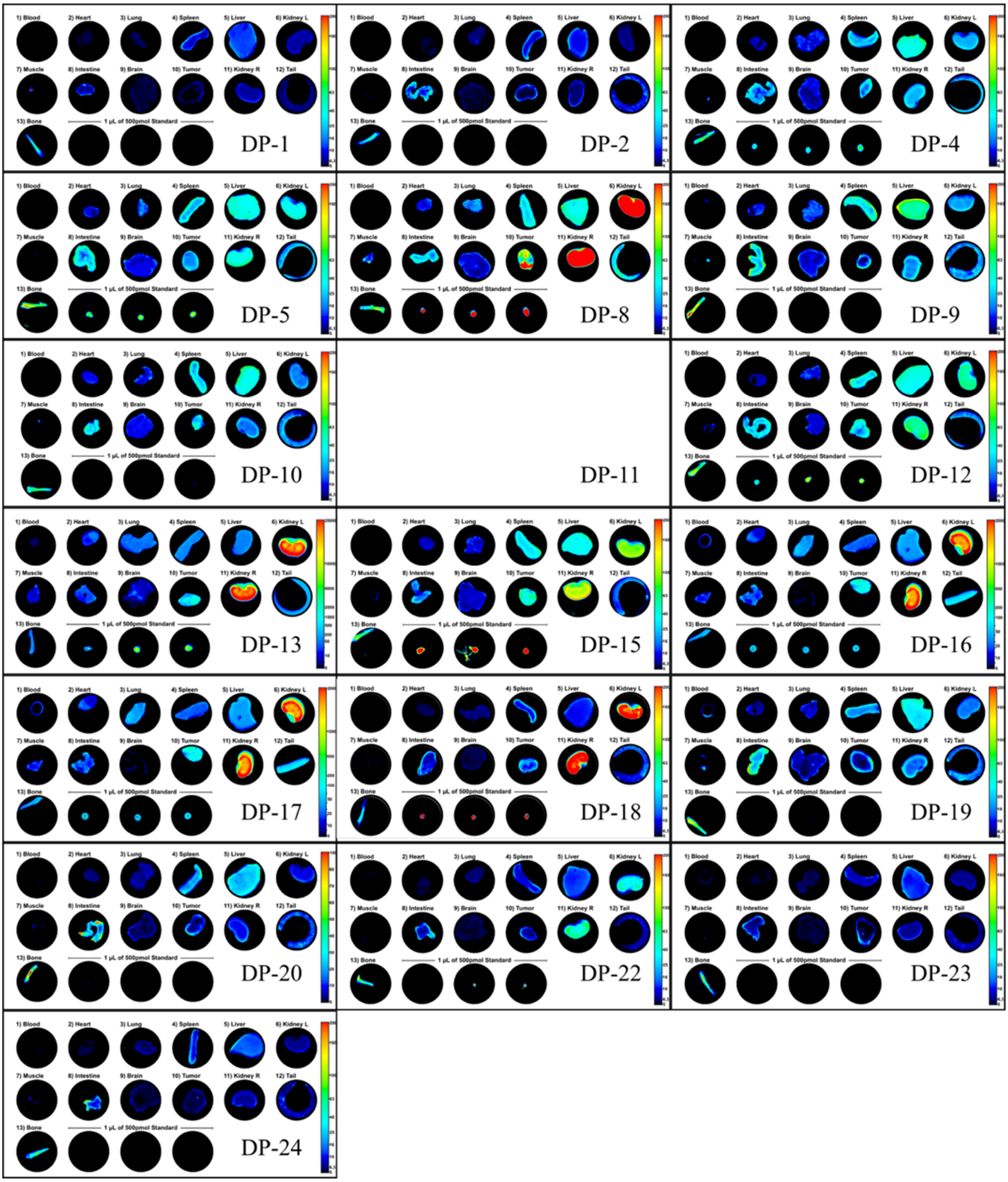

Supplementary Figure S 9: PSMA specificity confirmed in PC-3 (PSMA-negative) xenograft model. Following administration of 500 pmol ^{68}Ga-labeled compound of DP-13 (DP-IRDye800CW) in PC-3 tumor-bearing BALB/c nude mice. (A) PET/MR maximum intensity projection (MIP) images at 1 h & 2 h p.i., (B) fluorescence imaging (excitation 637 nm) of kidney (positive control), muscle (negative control), and PC-3 tumor, (C) radioactive organ distribution (%ID/g) at 2 h p.i., and (D) *ex vivo* fluorescence imaging using LI-COR Odyssey system, measuring at 800 nm.

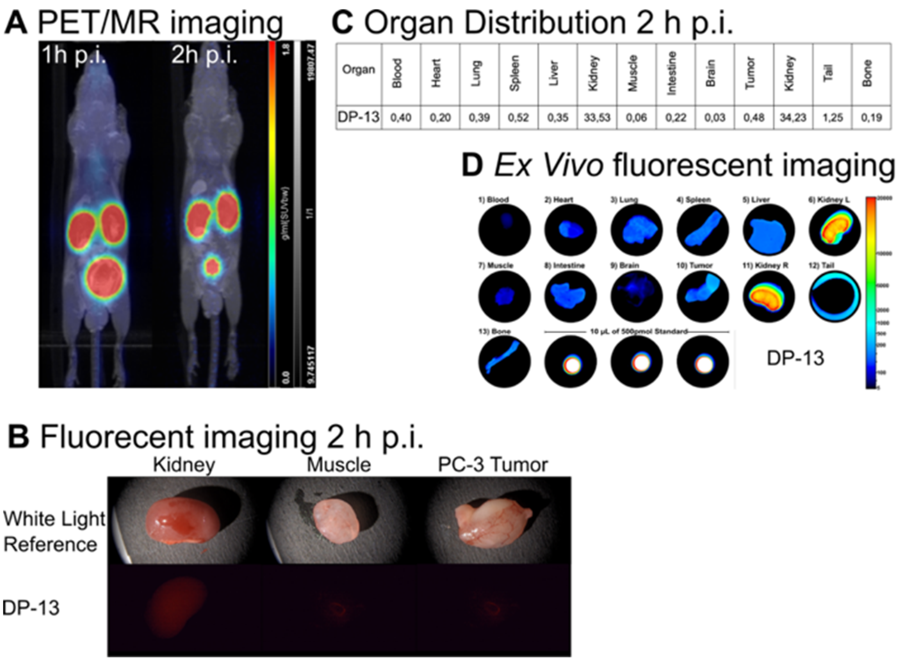

Supplement: Supplementary file 1 [file pharmaceuticals-19-01187-s001.zip › pharmaceuticals-4398186-supplementary.pdf]
